# TeloComp: An efficient toolkit for accurate assembly of the telomeres in T2T genomes

**DOI:** 10.1016/j.xplc.2025.101492

**Published:** 2025-08-23

**Authors:** Shou-Bian Huang, Jie Wu, Zi-Jian Xu, Wen-Tong Mo, Shuai Yuan, Xiao-Yao Jiang, Hai-Feng Wang, Liang Xie

**Affiliations:** 1 State Key Laboratory for Conservation and Utilization of Subtropical Agro-Bioresources, College of Agriculture, Guangxi University, Nanning 530004, China; 2 Key Laboratory of Crop Cultivation and Physiology, Education Department of Guangxi Zhuang Autonomous Region, Guangxi University, Nanning 530004, China; 3 Yazhouwan National Laboratory, Sanya, Hainan 572025, China

Dear Editor,

The completeness and accuracy of genome assemblies are crucial for ensuring the reliability of downstream analyses, including functional and evolutionary studies. With the advent of third-generation sequencing, the assembly of telomere-to-telomere (T2T) genomes has become possible. Telomeres are regions of tandemly repeated sequences at the ends of chromosomes that form a complex nucleoprotein structure together with protective proteins that act as a protective cap for the chromosome ends and maintain the integrity of the genome (Lyčka et al., 2024). However, because they are located at the ends of sequences and are highly complex, telomeres are often overlooked during whole-genome assembly, leading to assembly errors (such as the loss of telomeric repeat sequences in T2T genomes) that pose significant challenges for genome evolution studies.

Current tools such as Quartet, TelomereHunter, and EdgeCase (Feuerbach et al., 2019; Grigorev et al., 2021; Lin et al., 2023) can identify telomere sequences in existing genomes but cannot integrate sequencing data to complete unrecognized telomere regions (Supplemental Table 1). To address this gap, we developed TeloComp, an efficient and user-friendly integrated toolkit for telomere detection and complementation.

TeloComp is a sequencing-data-based software tool designed for the completion of telomere ends. It can supplement the minimum required number of covered reads for telomeres according to the user’s specific needs, assemble telomeres as completely as possible on the basis of existing data, and enhance the integrity of T2T genome assemblies. A schematic of the TeloComp workflow is shown in Figure 1A. TeloComp consists of four modules, which can be run separately for different genomes or executed as an integrated process. It is also compatible with multicore parallel operation, which can greatly facilitate pan-genome complementation. We also collected extensive telomere information from TeloBase (Lyčka et al., 2024) and found that plant telomere sequences are highly diverse, with the majority composed predominantly of TTTAGGG or CCCTAAA (Supplemental Figures 8 and 9). This work laid the foundation for our subsequent selection of telomere sequences. First, we aligned third-generation data, such as HiFi and ONT reads, to the genome and filtered out reads that extended beyond the chromosome ends. Next, we used species-specific telomere repeat motifs to identify sequences that required telomere assembly. We then used the overlap map method in Flye to further assemble these sequences by splicing them into smaller fragments at the left and right ends, resulting in complementary draft telomere sequences. In the third step, we polished these draft sequences using whole-genome sequencing data, re-aligned them to the genome, and extended the original genome to generate a genome sequence with complete telomeres. Finally, we quantified the length of each chromosome-end complement, identified the types of repetitive motifs in the telomeres, and visualized the final telomere-complementation results.

**Figure 1:**
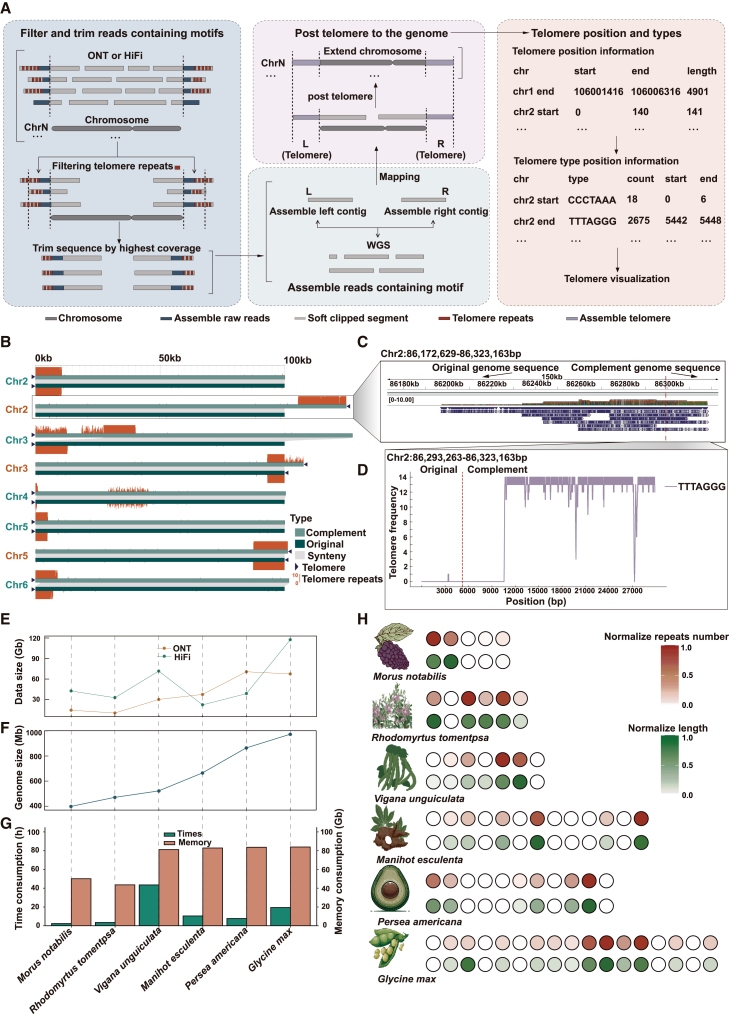
TeloComp workflow and its application for telomere completion. **(A)** TeloComp workflow diagram. (1) Input of genome data, followed by alignment, filtering, and extraction of reads with the corresponding coverage. (2) Assembly of the processed reads and output of the polished results. (3) Extraction of telomeric sequences, followed by their integration into the original genome and output of the genome with telomere complements. (4) Output of telomere positions, lengths, and counts, and visualization of results, including telomere density and synteny. **(B)** Collinearity alignment of *M. notabilis* in telomere regions. The 100-kb sequences are identical, and the extended regions represent the supplemented telomere sequences. Telomere repeat counts were calculated in 100-bp windows. Orange and cyan indicate the right and left ends of the chromosomes, respectively. Chromosome ends without supplemented telomere sequences are not shown. **(C)** The number of ONT reads supporting the 150-kb region at the right end of *M. notabilis* chromosome 2; the original genome and the supplemented telomere sequence are separated by a red dashed line (86 298 228 bp). **(D)** Density distribution of telomeric repeat monomers in the 30-kb region at the right end of *M. notabilis* chromosome 2 calculated using a 100-bp window. **(E–G)** Data from T2T genomes of six species and TeloComp benchmarking tests, with data from each species connected by dashed lines. Amount of ONT and HiFi data **(E)**, genome size **(F)**, and TeloComp runtime and memory usage **(G)**. **(H)** Lengths and numbers of supplemented telomere sequences in different genomes. The normalized number of sequences is shown in the top row, and the lengths are shown in the bottom row. The corresponding chromosome numbers are arranged in ascending order, and only chromosomes with supplemented telomeres are shown.

To evaluate the performance of TeloComp, we downloaded the T2T genome of *Morus notabilis* (398 Mb) from N3database (Xie et al., 2024). This genome consists of six chromosomes, but no telomeric sequences were originally detected at the right ends of chr1 and chr2 (Ma et al., 2023). We hypothesized that this absence might be due to gaps in the assembly of complex terminal regions. We first identified the telomeric sequences of *M. notabilis* as CCCTAAA and TTTAGGG using TeloBase (Supplemental Figure 9). Next, we ran TeloComp for telomere completion and found that a 24-kb DNA sequence was added to the right end of chr2 (Figure 1B). This sequence was supported by continuous ONT reads in the genome browser (Figure 1C), confirming the reliability of the extended region. Further analysis of the extended sequence revealed the presence of the repeating monomer TTTAGGG, which is consistent with the telomeric monomer characteristics of *M. notabilis*, indicating that the sequence completed by TeloComp was composed primarily of telomeric repeats (Figure 1D). In addition to the fully completed telomere on chr2, other chromosome ends were also extended to various degrees by both gap filling and telomere elongation. These results demonstrate that TeloComp can specifically capture chromosome-end sequences and accurately integrate them into the original genome, effectively addressing telomere loss in standard genome assemblies. As a result, TeloComp significantly improves the completeness of telomere assembly in T2T genomes (Figure 1B; Supplemental Table 3).

To evaluate the stability of TeloComp across genomes with various degrees of complexity and sequencing depths, we applied it to a broad range of plant lineages, including algae, bryophytes, ferns, angiosperms, gymnosperms, and monocots, as well as polyploid and large-genome species (Supplemental Tables 2, 4, and 5). Among angiosperm genomes, that of *Manihot esculenta* (Xu et al., 2023) showed the greatest improvement, with telomere extensions of 6.3–8.8 kb at the ends of chr1, chr7, chr8, and chr16 (Supplemental Figure 10C; Supplemental Table 3). Moderate telomere extension and supplementation were also observed in *Rhodomyrtus tomentosa*, *Vigna unguiculata* (Yang et al., 2023), *Persea americana* (Yang et al., 2024), and *Glycine max* (Zhang et al., 2023) (Supplemental Figure 10A, 10B, 10D, and 10E; Supplemental Table 3). In *Chlorella pyrenoidosa* DBH and *Physcomitrella patens*, TeloComp successfully completed telomere structures (Supplemental Figures 12 and 13; Supplemental Table 6). In *Oryza sativa* ssp. *japonica*, telomeres were restored on nearly all chromosomes, with extensions exceeding 1 kb on chr2, chr4, chr8, and chr10 (Supplemental Figure 15; Supplemental Table 6). For the large-genome species *Chlamydomonas reinhardtii*, TeloComp restored multiple telomeric regions, including a 9-kb extension of chr15 (Supplemental Figure 14; Supplemental Table 6). Although only a chromosome-level genome assembly was available for *Taxus chinensis* var. *mairei*, TeloComp identified and supplemented telomeres using the known CCCTAAA motif (Supplemental Figure 18; Supplemental Table 6). In polyploid genomes such as triploid *Musa acuminata × Musa balbisiana* and tetraploid *Nicotiana benthamiana*, telomere supplementation was also evident, with multiple chromosomes showing >1-kb extensions and a clear increase in telomere number (Supplemental Figures 16 and 17; Supplemental Table 6). These results demonstrate that TeloComp exhibits broad applicability and robust performance across diverse phylogenetic groups, ploidy levels, and genome sizes, with a particular strength in improving telomere completeness. This improvement greatly enhances the quality of genome assemblies and provides a solid foundation for investigating the evolution and function of telomeric regions.

We evaluated the computational efficiency of TeloComp by measuring the time and memory required to process six genomes, including that of *M. notabilis* (Figure 1G; Supplemental Table 2). The amount of third-generation sequencing data (HiFi and ONT) increased with genome size (Figure 1E and 1F), which reflected species genetic complexity. *R. tomentosa* and *M. notabilis* required the least time and memory (about 10 h) for telomere reconstruction, owing to their smaller genomes and less complex data. *V. unguiculata* required more time because of the larger amount of ONT data. For the larger genomes (cassava, avocado, and *G. max*), runtime was approximately 20 h but could be reduced by using pre-processed comparison results (Figure 1G). These results highlight the efficiency and adaptability of TeloComp across genomes of various complexities.

Telomeric regions, with their highly repetitive structural units, pose significant challenges for sequencing, filtering, and assembly. We also counted the increased number of telomeric motifs at the chromosome ends after running TeloComp on different genomes (Supplemental Table 3; Figure 1H). There were significant increases in both the number of telomeres and terminal sequences across the different genomes, with longer supplemented telomeres showing greater enrichment. The quality of telomere supplementation using TeloComp was highest in *M. notabilis* and cassava (Figure 1H; Supplemental Figure 11). These findings suggest that TeloComp’s performance is influenced by the inherent chromosome structure, assembly quality, and quality of assembly data when handling different genomes.

In summary, TeloComp is the first software specifically developed for telomere-assisted genome assembly. It introduces an efficient telomere-completion strategy based on existing sequencing data and is particularly effective in low-coverage and highly repetitive regions. To ensure accurate telomere assembly, it uses a unique read-filtering strategy that considers genome-wide coverage and the original genome alignment ratio. It also includes a dedicated assembly module designed to address the repetitive nature of telomeric regions, ensuring the completeness and precision of the assembled sequences. Its modular structure and integrated visualization tools enable users to flexibly assess and compare assembly outcomes across different strategies, making it a valuable tool for assembling telomeric regions in large and complex genomes. TeloComp is freely available at https://github.com/lxie-0709/TeloComp.git. We anticipate that TeloComp will complement experimental approaches such as fluorescence *in situ hybridization*, PCR, and telomere-specific fluorescence methods to provide a more comprehensive solution for telomere assembly and T2T genome research.

## Data availability

The source code of TeloComp is available on GitHub (https://github.com/lxie-0709/TeloComp.git).

## Funding

This work was supported by the 10.13039/501100001809National Natural Science Foundation of China (NSFC) (no. 32300475), the Science and Technology Talent Special Project of Guangxi (Gui Ke AD23026320), funding from the State Key Laboratory for Conservation and Utilization of Subtropical Agro-bioresources (SKLCUSA-b202307), and the Starting Research Grant for High-level Talents from 10.13039/501100012253Guangxi University to L.X.

## Acknowledgments

No conflict of interest is declared.

## Author contributions

L.X. conceived and designed the project. L.X. and S.-B.H. developed the TeloComp software. S.-B.H. collected the data and performed the analyses. S.-B.H., J.W., Z.-J.X., W.-T.M., S.Y., and X.-Y.J. mainly performed software usage testing. L.X., H.-F.W., and S.-B.H. wrote the paper. All authors read and approved the final manuscript.

## Supplemental information

Document S1. Supplemental Figures 1–18, Supplemental Tables 1–6, and supplemental methods

Document S2. Article plus supplemental information
