# Supplementary material for: TeloComp: An efficient toolkit for accurate assembly of the telomeres in T2T genomes
Source: Plant Commun. 2025 Aug 23;6(11):101492. doi: 10.1016/j.xplc.2025.101492 (PMC12785171; doi:10.1016/j.xplc.2025.101492)
Supplement: Document S2. Article plus supplemental information [file mmc2.pdf]

Plant Communications
Correspondence

CellPress
Partner Journal

TeloComp: An efficient toolkit for accurate assembly of the telomeres in T2T genomes

Dear Editor,

The completeness and accuracy of genome assemblies are crucial for ensuring the reliability of downstream analyses, including functional and evolutionary studies. With the advent of third-generation sequencing, the assembly of telomere-to-telomere (T2T) genomes has become possible. Telomeres are regions of tandemly repeated sequences at the ends of chromosomes that form a complex nucleoprotein structure together with protective proteins that act as a protective cap for the chromosome ends and maintain the integrity of the genome (Lyčka et al., 2024). However, because they are located at the ends of sequences and are highly complex, telomeres are often overlooked during whole-genome assembly, leading to assembly errors (such as the loss of telomeric repeat sequences in T2T genomes) that pose significant challenges for genome evolution studies.

Current tools such as Quartet, TelomereHunter, and EdgeCase (Feuerbach et al., 2019; Grigorev et al., 2021; Lin et al., 2023) can identify telomere sequences in existing genomes but cannot integrate sequencing data to complete unrecognized telomere regions (Supplemental Table 1). To address this gap, we developed TeloComp, an efficient and user-friendly integrated toolkit for telomere detection and complementation.

TeloComp is a sequencing-data-based software tool designed for the completion of telomere ends. It can supplement the minimum required number of covered reads for telomeres according to the user's specific needs, assemble telomeres as completely as possible on the basis of existing data, and enhance the integrity of T2T genome assemblies. A schematic of the TeloComp workflow is shown in Figure 1A. TeloComp consists of four modules, which can be run separately for different genomes or executed as an integrated process. It is also compatible with multicore parallel operation, which can greatly facilitate pan-genome complementation. We also collected extensive telomere information from TeloBase (Lyčka et al., 2024) and found that plant telomere sequences are highly diverse, with the majority composed predominantly of TTTAGGG or CCCTAAA (Supplemental Figures 8 and 9). This work laid the foundation for our subsequent selection of telomere sequences. First, we aligned third-generation data, such as HiFi and ONT reads, to the genome and filtered out reads that extended beyond the chromosome ends. Next, we used species-specific telomere repeat motifs to identify sequences that required telomere assembly. We then used the overlap map method in Flye to further assemble these sequences by splicing them into smaller fragments at the left and right ends, resulting in complementary draft telomere sequences. In the third step, we polished these draft sequences using whole-genome sequencing data, re-aligned them to the genome, and extended the original genome to generate a genome sequence with complete telomeres. Finally, we quantified the length of each chromosome-end complement, identified the types of repetitive motifs in the telomeres, and visualized the final telomere-complementation results.

To evaluate the performance of TeloComp, we downloaded the T2T genome of *Morus notabilis* (398 Mb) from N3database (Xie et al., 2024). This genome consists of six chromosomes, but no telomeric sequences were originally detected at the right ends of chr1 and chr2 (Ma et al., 2023). We hypothesized that this absence might be due to gaps in the assembly of complex terminal regions. We first identified the telomeric sequences of *M. notabilis* as CCCTAAA and TTTAGGG using TeloBase (Supplemental Figure 9). Next, we ran TeloComp for telomere completion and found that a 24-kb DNA sequence was added to the right end of chr2 (Figure 1B). This sequence was supported by continuous ONT reads in the genome browser (Figure 1C), confirming the reliability of the extended region. Further analysis of the extended sequence revealed the presence of the repeating monomer TTTAGGG, which is consistent with the telomeric monomer characteristics of *M. notabilis*, indicating that the sequence completed by TeloComp was composed primarily of telomeric repeats (Figure 1D). In addition to the fully completed telomere on chr2, other chromosome ends were also extended to various degrees by both gap filling and telomere elongation. These results demonstrate that TeloComp can specifically capture chromosome-end sequences and accurately integrate them into the original genome, effectively addressing telomere loss in standard genome assemblies. As a result, TeloComp significantly improves the completeness of telomere assembly in T2T genomes (Figure 1B; Supplemental Table 3).

To evaluate the stability of TeloComp across genomes with various degrees of complexity and sequencing depths, we applied it to a broad range of plant lineages, including algae, bryophytes, ferns, angiosperms, gymnosperms, and monocots, as well as polyploid and large-genome species (Supplemental Tables 2, 4, and 5). Among angiosperm genomes, that of *Manihot esculenta* (Xu et al., 2023) showed the greatest improvement, with telomere extensions of 6.3–8.8 kb at the ends of chr1, chr7, chr8, and chr16 (Supplemental Figure 10C; Supplemental Table 3). Moderate telomere extension and supplementation were also observed in *Rhodomyrtus tomentosa*, *Vigna unguiculata* (Yang et al., 2023), *Persea americana* (Yang et al., 2024), and *Glycine max* (Zhang et al., 2023) (Supplemental Figure 10A, 10B, 10D, and 10E; Supplemental Table 3). In *Chlorella pyrenoidosa* DBH and *Physcomitrella patens*, TeloComp successfully completed telomere structures (Supplemental Figures 12 and 13; Supplemental Table 6). In *Oryza sativa* ssp. *japonica*, telomeres were restored on nearly all chromosomes, with extensions exceeding 1 kb on chr2, chr4, chr8, and chr10 (Supplemental Figure 15; Supplemental Table 6). For the

This is an open access article under the CC BY license (http://creativecommons.org/licenses/by/4.0/).

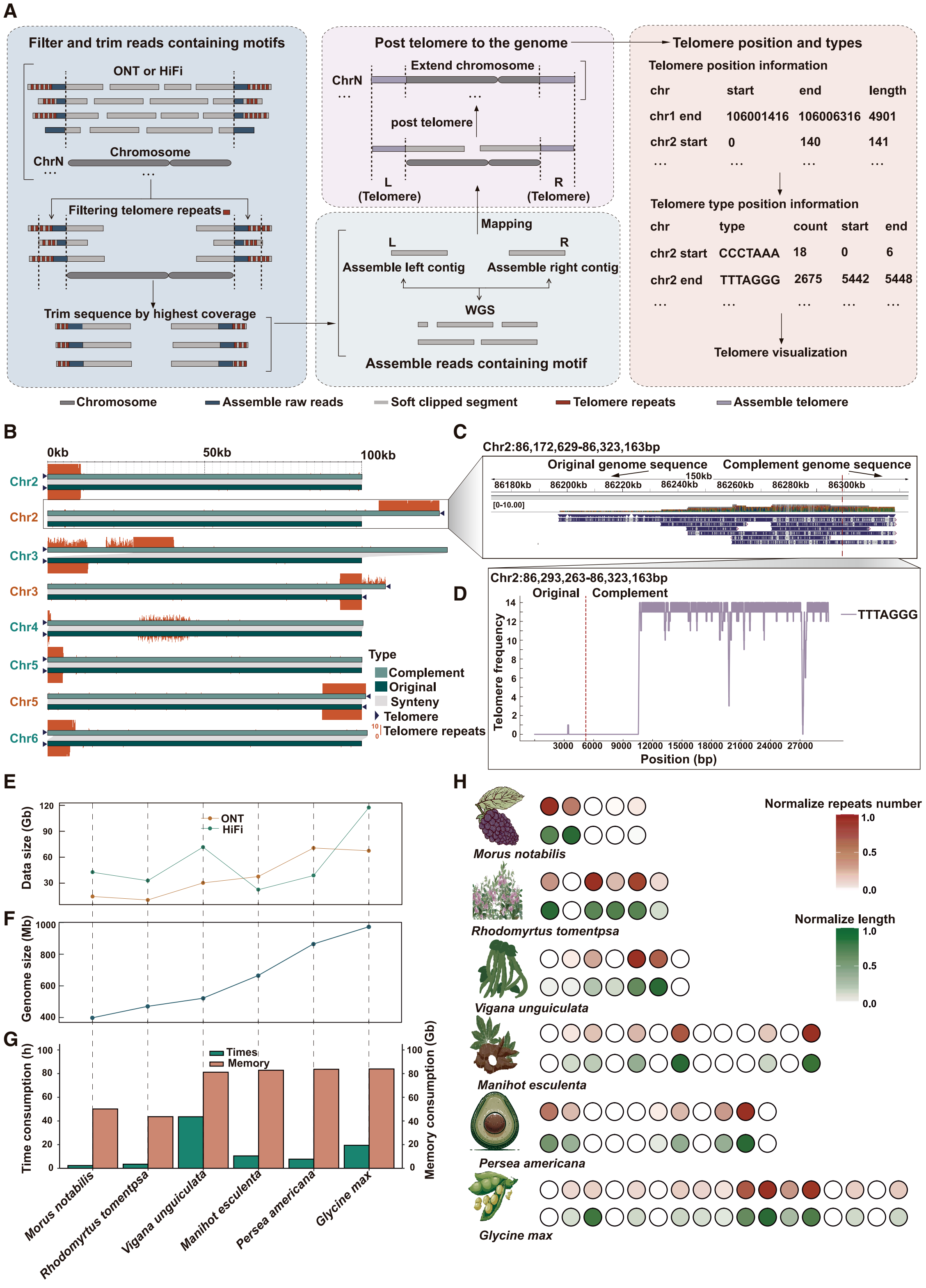

Figure 1. TeloComp workflow and its application for telomere completion.

(A) TeloComp workflow diagram. (1) Input of genome data, followed by alignment, filtering, and extraction of reads with the corresponding coverage. (2) Assembly of the processed reads and output of the polished results. (3) Extraction of telomeric sequences, followed by their integration into the original genome and output of the genome with telomere complements. (4) Output of telomere positions, lengths, and counts, and visualization of results, including telomere density and synteny.

(legend continued on next page)

large-genome species *Chlamydomonas reinhardtii*, TeloComp restored multiple telomeric regions, including a 9-kb extension of chr15 (Supplemental Figure 14; Supplemental Table 6). Although only a chromosome-level genome assembly was available for *Taxus chinensis* var. *mairei*, TeloComp identified and supplemented telomeres using the known CCCTAAA motif (Supplemental Figure 18; Supplemental Table 6). In polyploid genomes such as triploid *Musa acuminata* × *Musa balbisiana* and tetraploid *Nicotiana benthamiana*, telomere supplementation was also evident, with multiple chromosomes showing >1-kb extensions and a clear increase in telomere number (Supplemental Figures 16 and 17; Supplemental Table 6). These results demonstrate that TeloComp exhibits broad applicability and robust performance across diverse phylogenetic groups, ploidy levels, and genome sizes, with a particular strength in improving telomere completeness. This improvement greatly enhances the quality of genome assemblies and provides a solid foundation for investigating the evolution and function of telomeric regions.

We evaluated the computational efficiency of TeloComp by measuring the time and memory required to process six genomes, including that of *M. notabilis* (Figure 1G; Supplemental Table 2). The amount of third-generation sequencing data (HiFi and ONT) increased with genome size (Figure 1E and 1F), which reflected species genetic complexity. *R. tomentosa* and *M. notabilis* required the least time and memory (about 10 h) for telomere reconstruction, owing to their smaller genomes and less complex data. *V. unguiculata* required more time because of the larger amount of ONT data. For the larger genomes (cassava, avocado, and *G. max*), runtime was approximately 20 h but could be reduced by using pre-processed comparison results (Figure 1G). These results highlight the efficiency and adaptability of TeloComp across genomes of various complexities.

Telomeric regions, with their highly repetitive structural units, pose significant challenges for sequencing, filtering, and assembly. We also counted the increased number of telomeric motifs at the chromosome ends after running TeloComp on different genomes (Supplemental Table 3; Figure 1H). There were significant increases in both the number of telomeres and terminal sequences across the different genomes, with longer supplemented telomeres showing greater enrichment. The quality of telomere supplementation using TeloComp was highest in *M. notabilis* and cassava (Figure 1H; Supplemental Figure 11). These findings suggest that TeloComp's performance is influenced by the inherent chromosome structure, assembly quality, and quality of assembly data when handling different genomes.

In summary, TeloComp is the first software specifically developed for telomere-assisted genome assembly. It introduces an efficient telomere-completion strategy based on existing sequencing data and is particularly effective in low-coverage and highly repetitive regions. To ensure accurate telomere assembly, it uses a unique read-filtering strategy that considers genome-wide coverage and the original genome alignment ratio. It also includes a dedicated assembly module designed to address the repetitive nature of telomeric regions, ensuring the completeness and precision of the assembled sequences. Its modular structure and integrated visualization tools enable users to flexibly assess and compare assembly outcomes across different strategies, making it a valuable tool for assembling telomeric regions in large and complex genomes. TeloComp is freely available at https://github.com/lxie-0709/TeloComp.git. We anticipate that TeloComp will complement experimental approaches such as fluorescence *in situ hybridization*, PCR, and telomere-specific fluorescence methods to provide a more comprehensive solution for telomere assembly and T2T genome research.

DATA AVAILABILITY

The source code of TeloComp is available on GitHub (https://github.com/lxie-0709/TeloComp.git).

FUNDING

This work was supported by the National Natural Science Foundation of China (NSFC) (no. 32300475), the Science and Technology Talent Special Project of Guangxi (Gui Ke AD23026320), funding from the State Key Laboratory for Conservation and Utilization of Subtropical Agro-bioresources (SKLCUSA-b202307), and the Starting Research Grant for High-level Talents from Guangxi University to L.X.

ACKNOWLEDGMENTS

No conflict of interest is declared.

AUTHOR CONTRIBUTIONS

L.X. conceived and designed the project. L.X. and S.-B.H. developed the TeloComp software. S.-B.H. collected the data and performed the analyses. S.-B.H., J.W., Z.-J.X., W.-T.M., S.Y., and X.-Y.J. mainly performed software usage testing. L.X., H.-F.W., and S.-B.H. wrote the paper. All authors read and approved the final manuscript.

SUPPLEMENTAL INFORMATION

Supplemental information is available at *Plant Communications Online*.

(B) Collinearity alignment of *M. notabilis* in telomere regions. The 100-kb sequences are identical, and the extended regions represent the supplemented telomere sequences. Telomere repeat counts were calculated in 100-bp windows. Orange and cyan indicate the right and left ends of the chromosomes, respectively. Chromosome ends without supplemented telomere sequences are not shown.

(C) The number of ONT reads supporting the 150-kb region at the right end of *M. notabilis* chromosome 2; the original genome and the supplemented telomere sequence are separated by a red dashed line (86 298 228 bp).

(D) Density distribution of telomeric repeat monomers in the 30-kb region at the right end of *M. notabilis* chromosome 2 calculated using a 100-bp window.

(E–G) Data from T2T genomes of six species and TeloComp benchmarking tests, with data from each species connected by dashed lines. Amount of ONT and HiFi data **(E)**, genome size **(F)**, and TeloComp runtime and memory usage **(G)**.

(H) Lengths and numbers of supplemented telomere sequences in different genomes. The normalized number of sequences is shown in the top row, and the lengths are shown in the bottom row. The corresponding chromosome numbers are arranged in ascending order, and only chromosomes with supplemented telomeres are shown.

Received: November 9, 2024
Revised: April 28, 2025
Accepted: August 20, 2025
Published: August 23, 2025

Shou-Bian Huang[1,2]*, Jie Wu*[1,2]*, Zi-Jian Xu*[1,2]*, Wen-Tong Mo*[1,2]*, Shuai Yuan*[1,2]*, Xiao-Yao Jiang*[1,2]*, Hai-Feng Wang*[3,*] *and Liang Xie*[1,2,*]

[1]State Key Laboratory for Conservation and Utilization of Subtropical Agro-Bioresources, College of Agriculture, Guangxi University, Nanning 530004, China
[2]Key Laboratory of Crop Cultivation and Physiology, Education Department of Guangxi Zhuang Autonomous Region, Guangxi University, Nanning 530004, China
[3]Yazhouwan National Laboratory, Sanya, Hainan 572025, China
***Correspondence: Hai-Feng Wang (haifengwang@gxu.edu.cn), Liang Xie (lxie@gxu.edu.cn)**
https://doi.org/10.1016/j.xplc.2025.101492

REFERENCES

Feuerbach, L., Sieverling, L., Deeg, K.I., Ginsbach, P., Hutter, B., Buchhalter, I., Northcott, P.A., Mughal, S.S., Chudasama, P., Glimm, H., et al. (2019). TelomereHunter – in silico estimation of telomere content and composition from cancer genomes. BMC Bioinf. **20**:272. https://doi.org/10.1186/s12859-019-2851-0.

Grigorev, K., Foox, J., Bezdan, D., Butler, D., Luxton, J.J., Reed, J., McKenna, M.J., Taylor, L., George, K.A., Meydan, C., et al. (2021). Haplotype diversity and sequence heterogeneity of human telomeres. Genome Res. **31**:1269–1279. https://doi.org/10.1101/gr.274639.120.

Lin, Y., Ye, C., Li, X., Chen, Q., Wu, Y., Zhang, F., Pan, R., Zhang, S., Chen, S., Wang, X., et al. (2023). quarTeT: a telomere-to-telomere toolkit for gap-free genome assembly and centromeric repeat identification. Hortic. Res. **10**:uhad127. https://doi.org/10.1093/hr/uhad127.

Lyčka, M., Bubeník, M., Závodník, M., Peska, V., Fajkus, P., Demko, M., Fajkus, J., and Fojtová, M. (2024). TeloBase: a community-curated database of telomere sequences across the tree of life. Nucleic Acids Res. **52**:D311–D321. https://doi.org/10.1093/nar/gkad672.

Ma, B., Wang, H., Liu, J., Chen, L., Xia, X., Wei, W., Yang, Z., Yuan, J., Luo, Y., and He, N. (2023). The gap-free genome of mulberry elucidates the architecture and evolution of polycentric chromosomes. Hortic. Res. **10**:uhad111. https://doi.org/10.1093/hr/uhad111.

Xie, L., Gong, X., Yang, K., Huang, Y., Zhang, S., Shen, L., Sun, Y., Wu, D., Ye, C., Zhu, Q.-H., and Fan, L. (2024). Technology-enabled great leap in deciphering plant genomes. Nat. Plants **10**:551–566. https://doi.org/10.1038/s41477-024-01655-6.

Xu, X.-D., Zhao, R.-P., Xiao, L., Lu, L., Gao, M., Luo, Y.-H., Zhou, Z.-W., Ye, S.-Y., Qian, Y.-Q., Fan, B.-L., et al. (2023). Telomere-to-telomere assembly of cassava genome reveals the evolution of cassava and divergence of allelic expression. Hortic. Res. **10**:uhad200. https://doi.org/10.1093/hr/uhad200.

Yang, T., Cai, Y., Huang, T., Yang, D., Yang, X., Yin, X., Zhang, C., Yang, Y., and Yang, Y. (2024). A telomere-to-telomere gap-free reference genome assembly of avocado provides useful resources for identifying genes related to fatty acid biosynthesis and disease resistance. Hortic. Res. **11**:uhae119. https://doi.org/10.1093/hr/uhae119.

Yang, Y., Wu, Z., Wu, Z., Li, T., Shen, Z., Zhou, X., Wu, X., Li, G., and Zhang, Y. (2023). A near-complete assembly of asparagus bean provides insights into anthocyanin accumulation in pods. Plant Biotechnol. J. **21**:2473–2489. https://doi.org/10.1111/pbi.14142.

Zhang, C., Xie, L., Yu, H., Wang, J., Chen, Q., and Wang, H. (2023). The T2T genome assembly of soybean cultivar ZH13 and its epigenetic landscapes. Mol. Plant **16**:1715–1718. https://doi.org/10.1016/j.molp.2023.10.003.

Plant Communications, Volume *6*

Supplemental information

TeloComp: An efficient toolkit for accurate assembly of the telomeres in T2T genomes

Shou-Bian Huang, Jie Wu, Zi-Jian Xu, Wen-Tong Mo, Shuai Yuan, Xiao-Yao Jiang, Hai-Feng Wang, and Liang Xie

Supplemental information

TeloComp: an efficient toolkit for accurate assembly of the telomeres in T2T genome

Shou-Bian Huang[1,2], Jie Wu[1,2], Zi-Jian Xu[1,2], Wen-Tong Mo[1,2], Shuai Yuan[1,2], Xiao-Yao Jiang[1,2], Hai-Feng Wang[3*], and Liang Xie[1,2*]

[1]State Key Laboratory for Conservation and Utilization of Subtropical Agro-Bioresources, College of Agriculture, Guangxi University, Nanning 530004, China;

[2]Key Laboratory of Crop Cultivation and Physiology, Education Department of Guangxi Zhuang Autonomous Region, Guangxi University, Nanning 530004, China;

[3]Yazhouwan National Laboratory, Sanya, Hainan 572025, China.

[*]Correspondence: lxie@gxu.edu.cn (L.X.) and haifengwang@gxu.edu.cn (H.W.)

This file includes:

1.Supplemental methods

2.Supplemental figures 1-18

3. Legend of Supplemental Tables

Supplemental methods

Comparative analysis of TeloComp and traditional telomere-related tools

Currently, traditional telomere-related tools have considerable limitations (Supplemental Table 1). For example, Teloclip merely filters terminal reads containing telomeric motifs; it lacks both telomere identification functionality and any assembly or scaffolding steps. Other telomere detection tools such as TIDK, quartet, TelomereHunter, and edgecase only quantify telomeric repeat sequences in fully assembled genomes to determine whether chromosomes contain telomere sequences. However, they do not utilize existing read data to perform telomeric sequence assembly or extension (Supplemental Table 1).

Due to the highly repetitive nature of telomeric regions and their terminal positioning, conventional genome assemblers often miss specific reads with low coverage and high repetitiveness during initial assembly, leading to telomere loss and compromised genome completeness. Acknowledging the limitations of current tools and the increasing need for telomere-to-telomere (T2T) genome assemblies, we developed TeloComp to fill this gap and improve the assambly of telomeric sequences.

TeloComp install and dependencies

The TeloComp software is designed and executed using Python version 3.11.6. It requires the following software: samtools-1.18 (Danecek et al., 2021), minimap2-2.27 (Li, 2018), bwa-0.7.17 (Li and Durbin, 2009), Flye-2.9.4 (Kolmogorov et al., 2019), Pilon version 1.24(Walker et al., 2014), NextPolish-v1.4.1 (Hu et al., 2019) and GenomeSyn-1.2.7 (Zhou et al., 2022). For installation instructions for TeloComp, please visit https://github.com/lxie-0709/TeloComp.git.

The principles and workflow of TeloComp

Data Input: For each species, TeloComp requires the input of genome data, ONT or HiFi sequencing data, whole Genome Sequencing data, and telomere motifs. Specifically, ONT and HiFi sequencing data can be provided as input to telocomp_Filter_1 to generate the BAM files required for downstream analysis. The command-line parameters should be specified as "--genome genome --fai genome.fai --ont ONT --hifi HiFi". If the corresponding BAM files have

already been generated (e.g., by aligning ONT or HiFi reads to the reference genome), telocomp_Filter_2 can be used directly to perform the initial filtering step. The command-line parameters for this step should be specified as "--ont_bam ont.bam --hifi_bam hifi.bam -o output_dir -c coverage".

Filtering end reads with motifs

During the data quality control phase, TeloComp not only filters out basic telomeric sequences but also removes reads extending beyond the chromosomal ends, retaining only those whose aligned portion covers ≥20% of the read length when mapped to the reference genome (Supplemental Figure 2). This threshold helps ensure that selected reads are correctly mapped to the chromosomal termini, particularly in highly repetitive telomeric regions, thereby minimizing assembly errors and improving the accuracy and reproducibility of the final assembly.

The threshold setting is based on biological rationale and systematic experimental evaluation, with the following details: telomeric reads typically consist of telomeric repeats and adjacent non-telomeric regions, especially near the telomere-subtelomere boundary. Retaining only reads composed almost entirely of telomeric repeats (i.e., those with minimal or no alignment to the reference genome) may result in the loss of crucial anchoring information, affecting the accurate reconstruction of chromosomal ends. Therefore, we adopted a relatively relaxed lower threshold to preserve these useful reads while minimizing the inclusion of erroneous alignments. We evaluated multiple candidate thresholds (10%, 20%, 30%, 40%, 50%, 60%, 70%, 80%), as shown in Supplemental Figure 1. The results indicate that thresholds between 10% and 40% enable most extension reads to be effectively integrated into the target regions. However, when the threshold exceeds 40%, the number of available reads drops sharply, complicating or preventing successful extension.

After a comprehensive balance among extension accuracy, read availability, and assembly feasibility, we selected 20% as the default threshold. This value is implemented in the software as a user-configurable parameter "--min_ratio", allowing users to flexibly adjust it according to the characteristics of their dataset.

For coverage control, TeloComp by default selects the shortest subset of filtered reads whose cumulative coverage reaches the maximum possible assembly value. Users can also manually define the desired coverage using the "-c / --coverage" parameter. If the assembly is successful, a continuous sequence (contig) is generated, and its corresponding telomeric sequence is re-integrated into the original genome (Supplemental Figure 2). During the filtering process, if only a single read is detected at a chromosome end, we evaluate whether its mapped portion covers at least 20% of the total read length (i.e., >10 kb mapped to the genome). If so, the telomeric sequence is extracted from this read and directly integrated into the genome; otherwise, the read is discarded to avoid introducing erroneous information (Supplemental Figure 2).

Based on this strategy, we first import the genome, ONT, HiFi, and telomeric motif files. ONT and HiFi reads are aligned to the genome using minimap2-2.27, and the results are converted to BAM format using samtools-1.18, retaining only those reads mapped to chromosomal ends. We then use teloclip-0.0.4 to extract reads containing telomeric motifs. The filtered reads are subsequently trimmed to a uniform length based on the maximum coverage (Figure 1A). Users may also specify a coverage range (20%–100%) for trimming. The final output includes two folders, "trim_L" and "trim_R", corresponding to the left and right telomeric ends, respectively, for downstream assembly and optimization. Additionally, if only the longest or shortest telomeric reads are required, users can extract high-quality reads directly using TeloComp's "telocomp_maxmin --Max_length" or "telocomp_maxmin --Min_length" parameters (without assembly), resulting in outputs labeled "MaxLength_NP" and "MinLength_NP" as the final assembled telomeric sequences (Supplemental Figure 2).

Assemble strategy

TeloComp provides a flexible telomeric overhang assembly framework that integrates both an internal Assemble module and the high-performance genome assembler Flye. The Assemble module is a lightweight Python-based tool designed to assemble a relatively small number of short telomeric reads. It emphasizes interpretability and customizability, allowing users to track the logical steps of local assembly and investigate structural differences.

Parameters such as --kmer_size, --min_overlap, and --error_rate enable fine-tuned adjustment to suit datasets of varying complexity.

In contrast, Flye is a high-performance assembler implemented in C++ that leverages an innovative repeat graph data structure. This architecture uses approximate sequence matching rather than strict k-mer exact matching, which effectively addresses the high noise levels commonly observed in long reads generated by single-molecule sequencing platforms such as PacBio and Oxford Nanopore Technologies. This approach enables Flye to resolve complex repetitive regions and produce high-quality assemblies, even under conditions of incomplete read coverage.

Unlike the Assemble module, which does not explicitly model repetitive regions and adopts a more aggressive assembly strategy suitable for simpler scenarios, Flye prioritizes assembly accuracy. It actively terminates extension in ambiguous or low-coverage regions to avoid introducing misassemblies. These algorithmic differences naturally lead to variation in the assembled overhang lengths across species. For example, the Assemble module tends to produce longer extensions, while Flye generates more conservative but biologically reliable results.

To assess whether these discrepancies arose from algorithmic design rather than assembly errors, we conducted genome-wide collinearity and homology analyses between the assembled overhangs and reference genomes (Supplementary Figure 3). Although the assembled lengths varied, the results remained highly collinear in both genomic location and functional annotation (Supplementary Figures 5A–7A). In scenarios where extension length differences were minimal, collinearity was nearly identical (Supplementary Figures 5B–7B). Additionally, assembly efficiency comparisons (Supplementary Figure 4) revealed that Flye significantly outperformed the Assemble module across all tested species, particularly for large datasets. This improvement is attributed to Flye’s repeat-aware graph structure and multithreaded optimization.

In practice, we used Flye v2.9.4 to assemble the trimmed telomeric reads generated in the previous step. For reads that were too short or of insufficient quality for successful assembly,

we adopted a fallback strategy that selects the shortest high-confidence read as the representative overhang to preserve key telomeric anchor information.

Subsequently, we performed polishing using both whole-genome sequencing (WGS) and HiFi reads. Preliminary correction was carried out using Pilon v1.24, followed by fine-scale polishing with NextPolish v1.4.1. Similar to the assembly stage, for reads that failed to be successfully corrected during polishing, the original uncorrected sequences were retained and included in the final output to ensure completeness and preserve biological relevance.

By default, TeloComp uses Flye as the primary assembly engine, balancing accuracy and efficiency. Meanwhile, the Assemble module is retained as an optional lightweight alternative, particularly useful for transparent interpretation or small-scale scenarios requiring localized telomere reconstruction.

Post telomere to the genome and Telomere position and types

We input the polished reads from the previous step, align them to the original genome using minimap2-2.27, and convert the alignment results to SAM format with samtools-1.18. Pysam-0.22.0 is used to parse the SAM file, and the positions of the telomeric regions are determined based on the start and end positions of the left and right ends, respectively. These telomeric regions are then extracted and added to the corresponding chromosome end.

Visualized results for TeloComp

TeloComp outputs detailed information about the positions and types of telomeres. Additionally, TeloComp calculates the telomere density distribution by dividing the genome into 100 kb windows and outputs the density distribution of telomeres and the top two other repetitive sequences. It does this by taking the 100 kb (Users can also set their own visualization length) region of the original genome near the telomere, as well as the region of the supplemented telomere, and compares the collinearity of these regions using GenomeSyn-1.2.7. This allows users to quickly visualize the newly added telomere segments and make adjustments based on the collinearity plots (Figure 1B; Supplemental Figure 3A-3E）. Users can customize how long the sequences are that will be extracted for visualization, adjusting them on a case-by-case basis.

Supplementary Figures

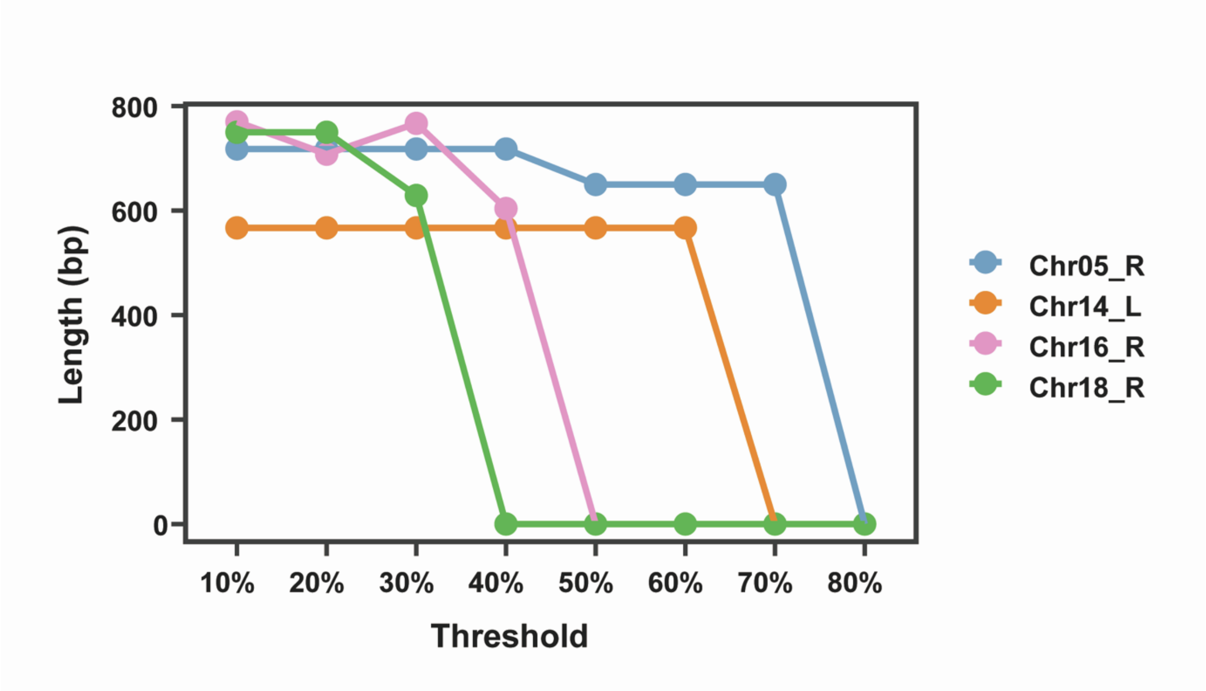

Supplemental Figure 1. Evaluation of *Nicotiana benthamiana* read assemblies under various threshold settings.

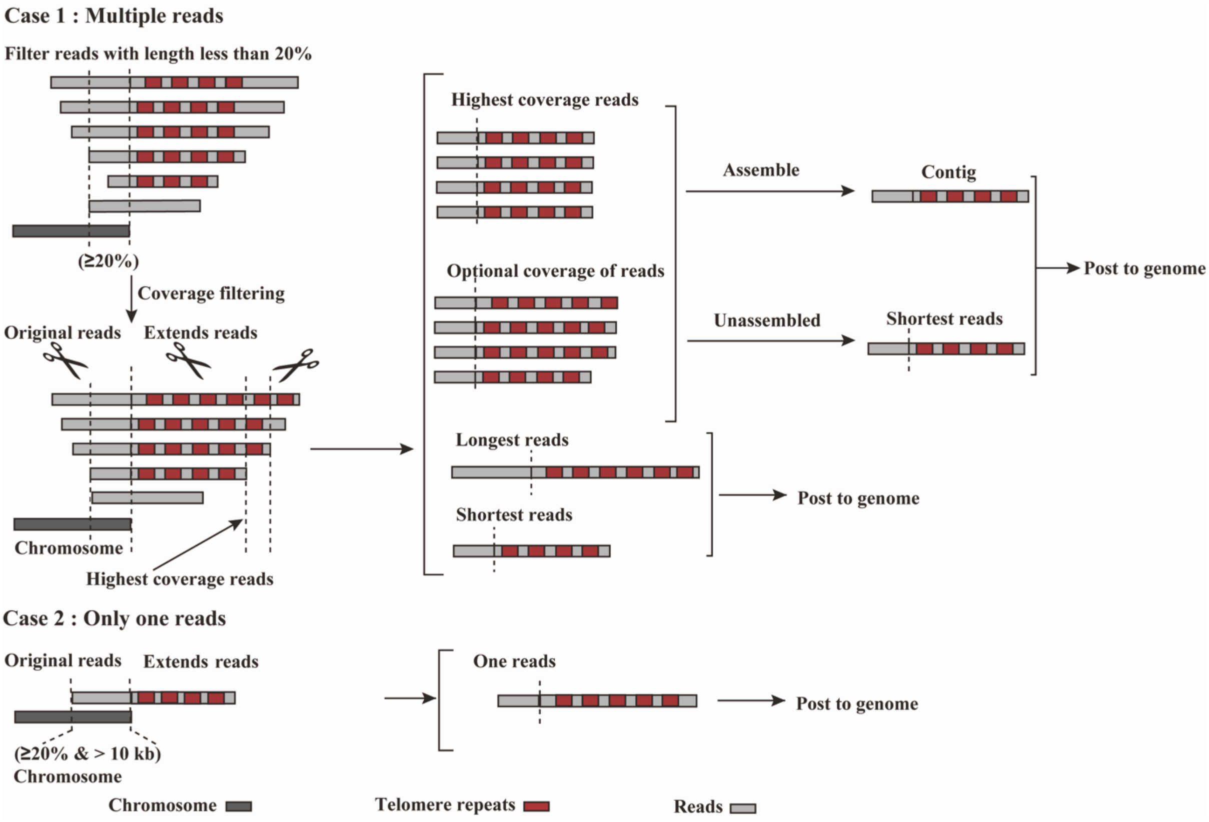

164 **Supplemental Figure 2. Schematic diagram illustrating read sorting and coverage**
165 **selection in TeloComp.**

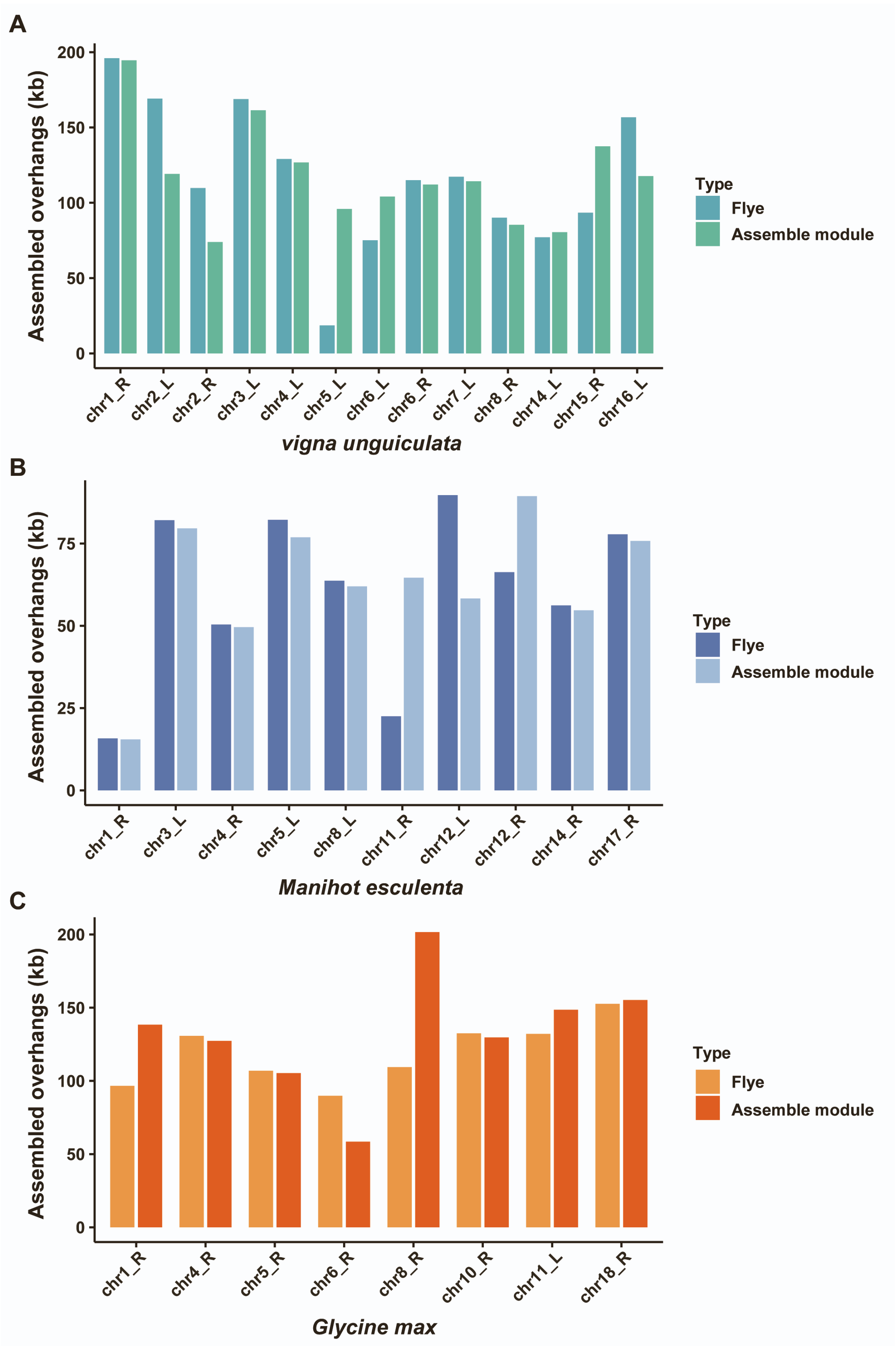

Supplemental Figure 3. Comparison of telomeric extension lengths assembled by Flye and the Assemble module.

(A-C) Length distributions of terminally extended regions assembled at chromosome ends in three plant species: (A) Vigna unguiculata (B) Manihot esculenta, and (C) Glycine max.

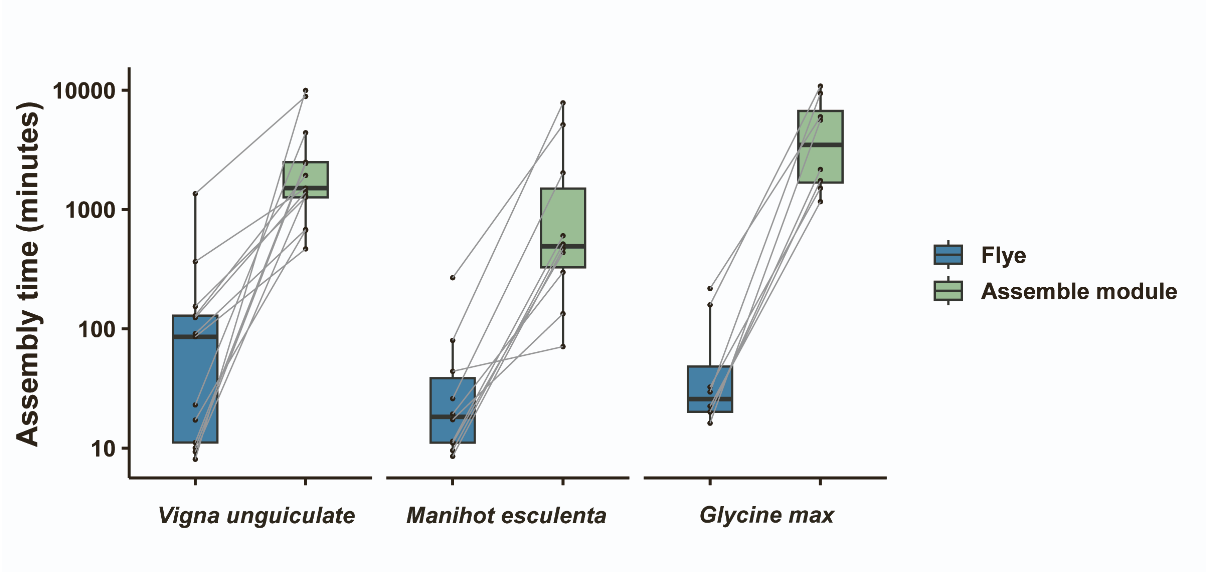

Supplemental Figure 4. Comparison of assembly speeds between Flye and assemble module.

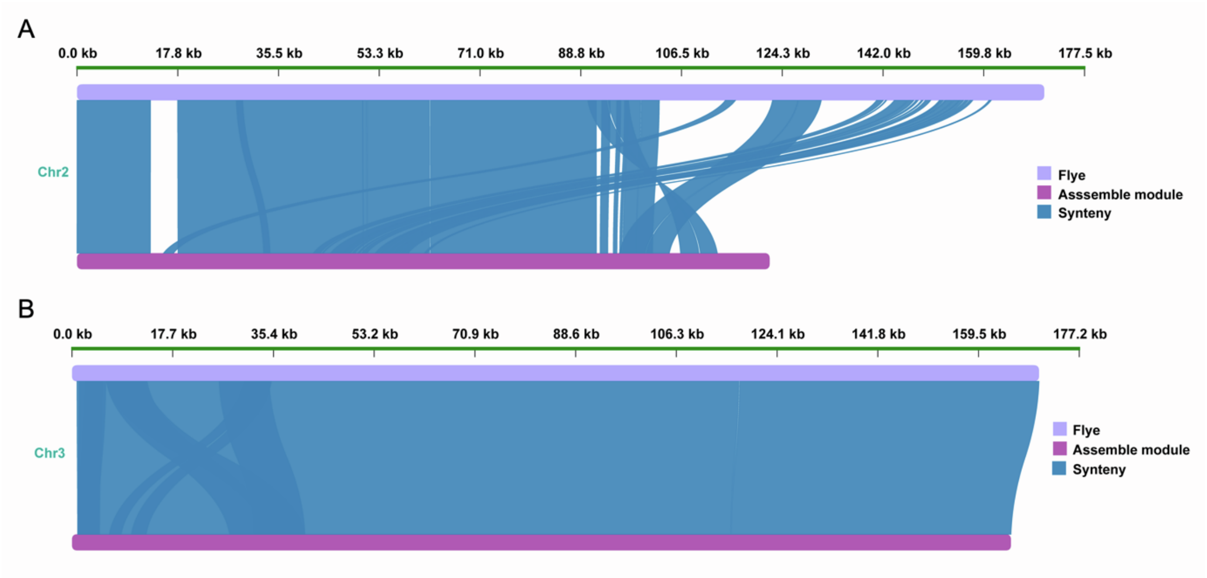

Supplemental Figure 5. Collinearity comparison of the left ends of *vigna unguiculata* chromosomes 2 and 3 assembled using Flye and the assemble module.

(A) Left end of *vigna unguiculata* chromosome 2 with a significant difference in assembly length.

(B) Left end of vigna unguiculata chromosome 3 with a minor difference in assembly length.

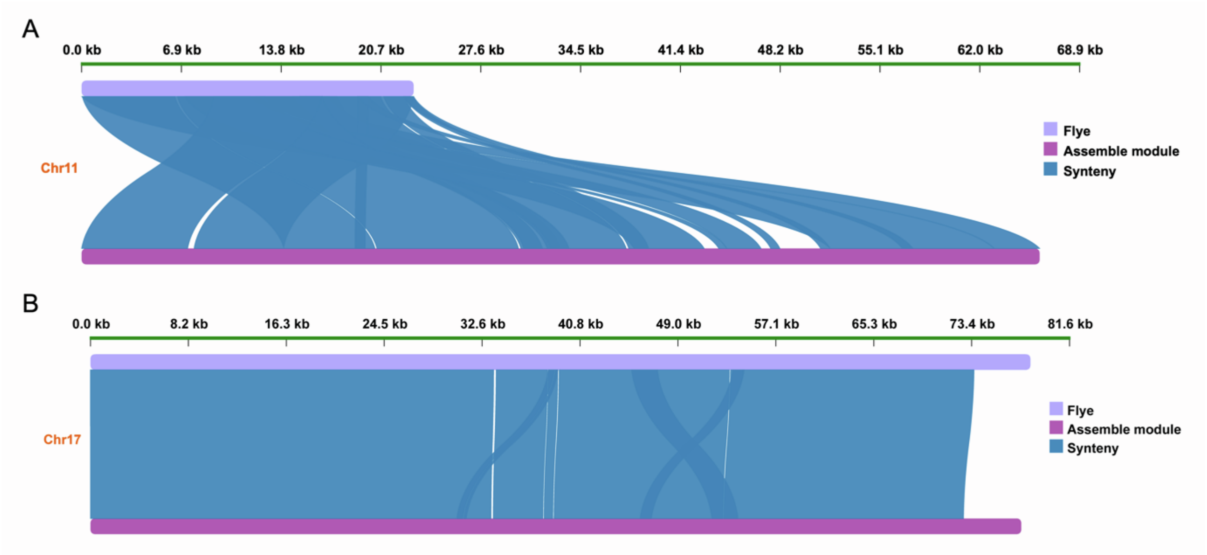

Supplemental Figure 6. Collinearity comparison of the right ends of *Manihot esculenta* chromosome 11 and chromosome 17 assembled using Flye and the assemble module.

(A) The right end of Manihot esculenta chromosome 11 with a significant difference in assembly length.

(B) The right end of Manihot esculenta chromosome 17 with a small difference in assembly length.

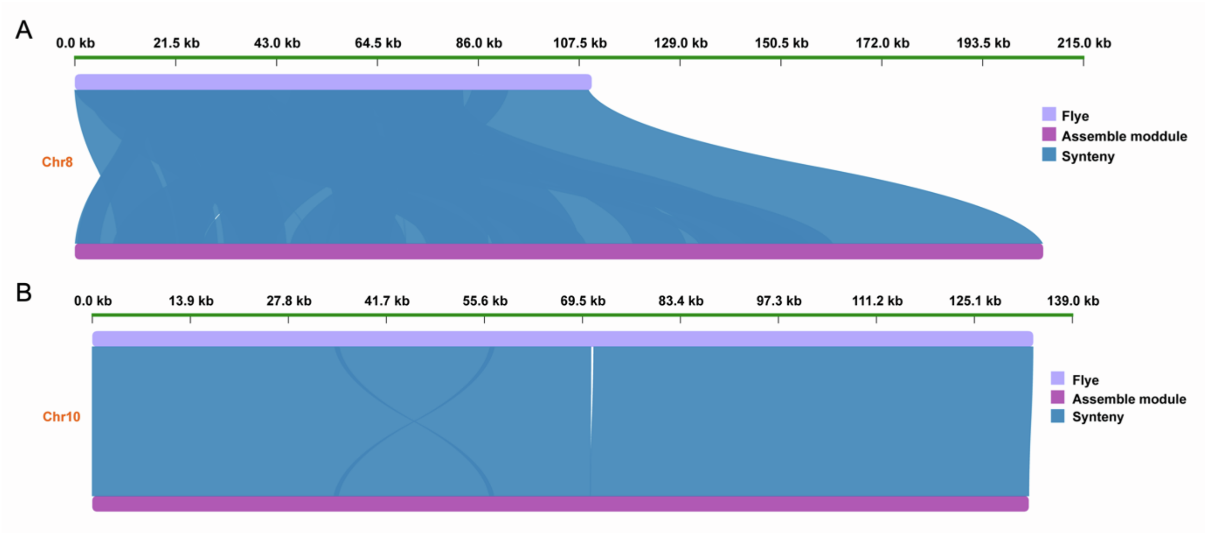

Supplemental Figure 7. Collinearity comparison of the right ends of *Glycine max* chromosome 8 and chromosome 10 assembled using Flye and the assemble module.

(A) The right end of Glycine max chromosome 8 with a significant assembly difference.

(B) The right end of Glycine max chromosome 10 with a similar assembly result.

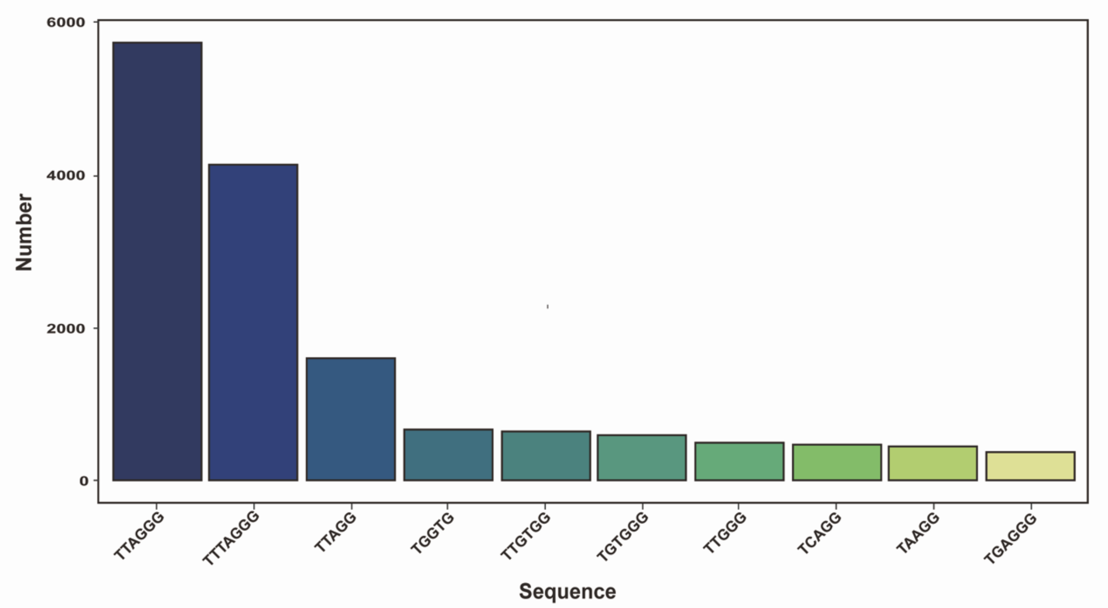

Supplemental Figure 8. The top 10 telomere types and quantity statistics in TeloBase.

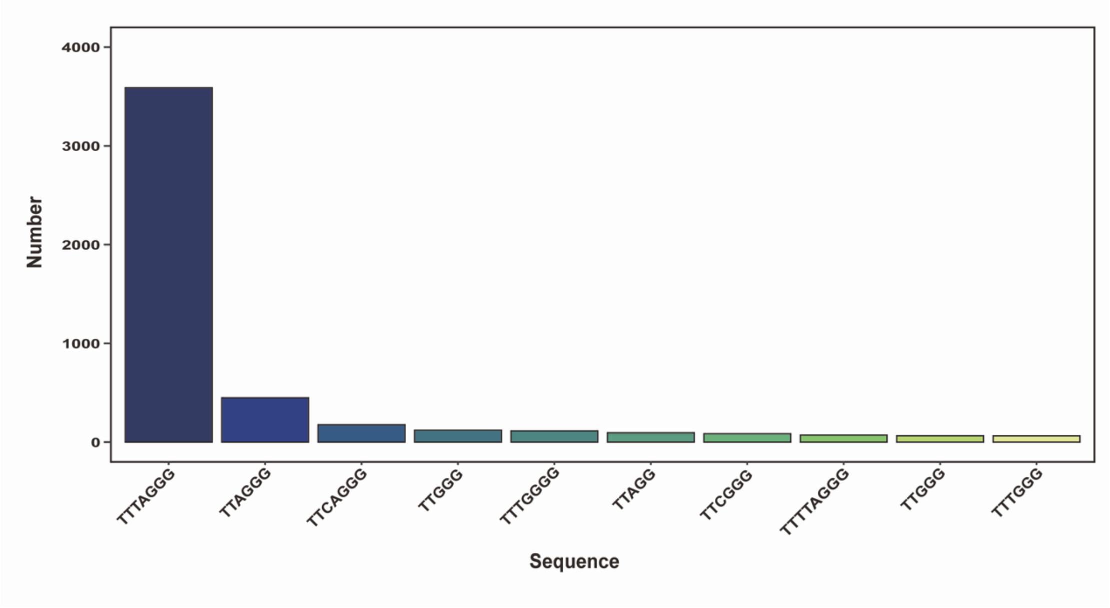

Supplemental Figure 9. The top 10 plant telomere types and quantity statistics in TeloBase.

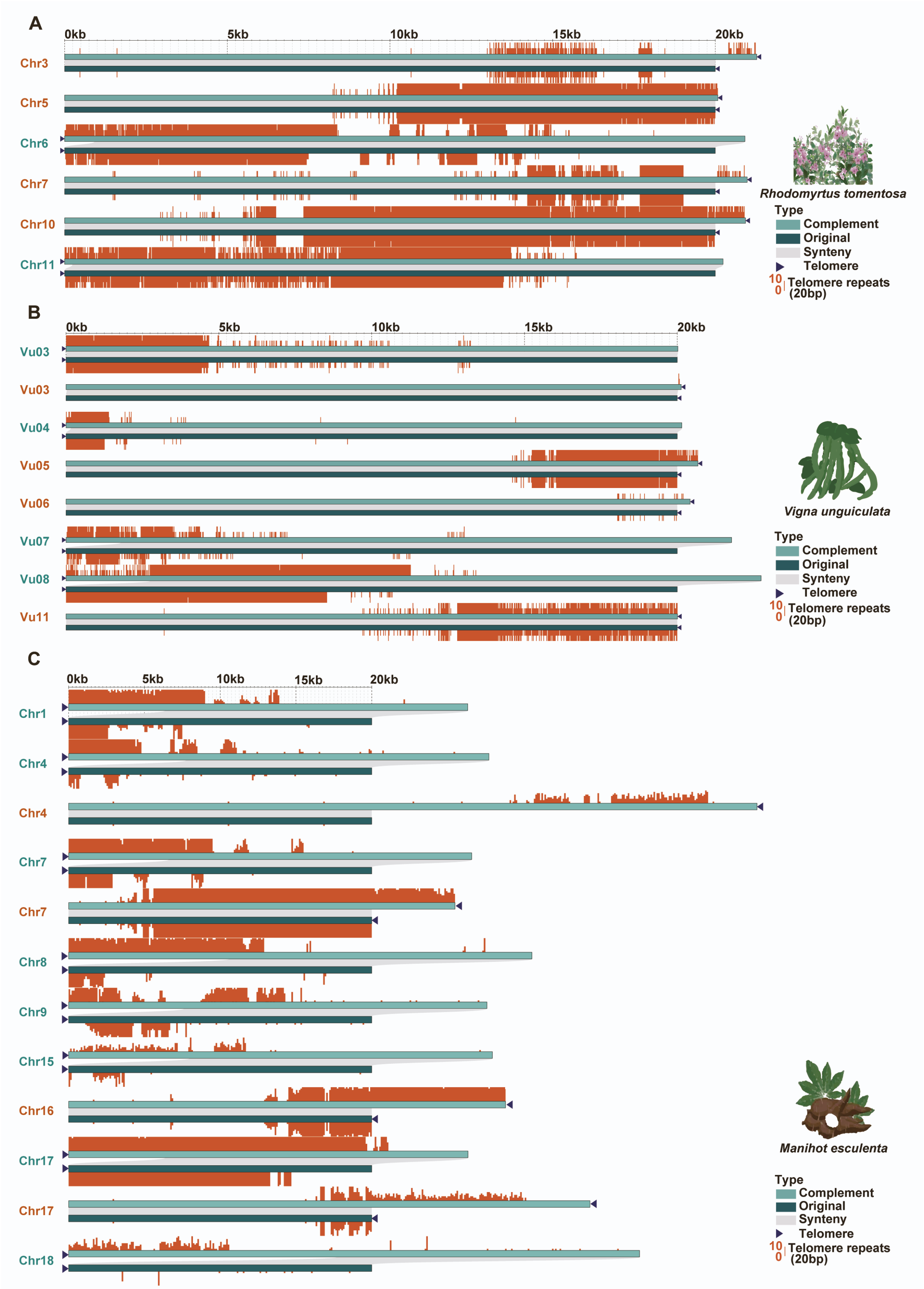
A
0kb
5kb
10kb
15kb
20kb
Chr3
Chr5
Chr6
Chr7
Chr10
Chr11
Rhodomyrtus tomentosa
Type
Complement
Original
Synteny
Telomere
10
0
Telomere repeats
(20bp)
B
0kb
5kb
10kb
15kb
20kb
Vu03
Vu03
Vu04
Vu05
Vu06
Vu07
Vu08
Vu11
Vigna unguiculata
Type
Complement
Original
Synteny
Telomere
10
0
Telomere repeats
(20bp)
C
0kb
5kb
10kb
15kb
20kb
Chr1
Chr4
Chr4
Chr7
Chr7
Chr8
Chr9
Chr15
Chr16
Chr17
Chr17
Chr18
Manihot esculenta
Type
Complement
Original
Synteny
Telomere
10
0
Telomere repeats
(20bp)

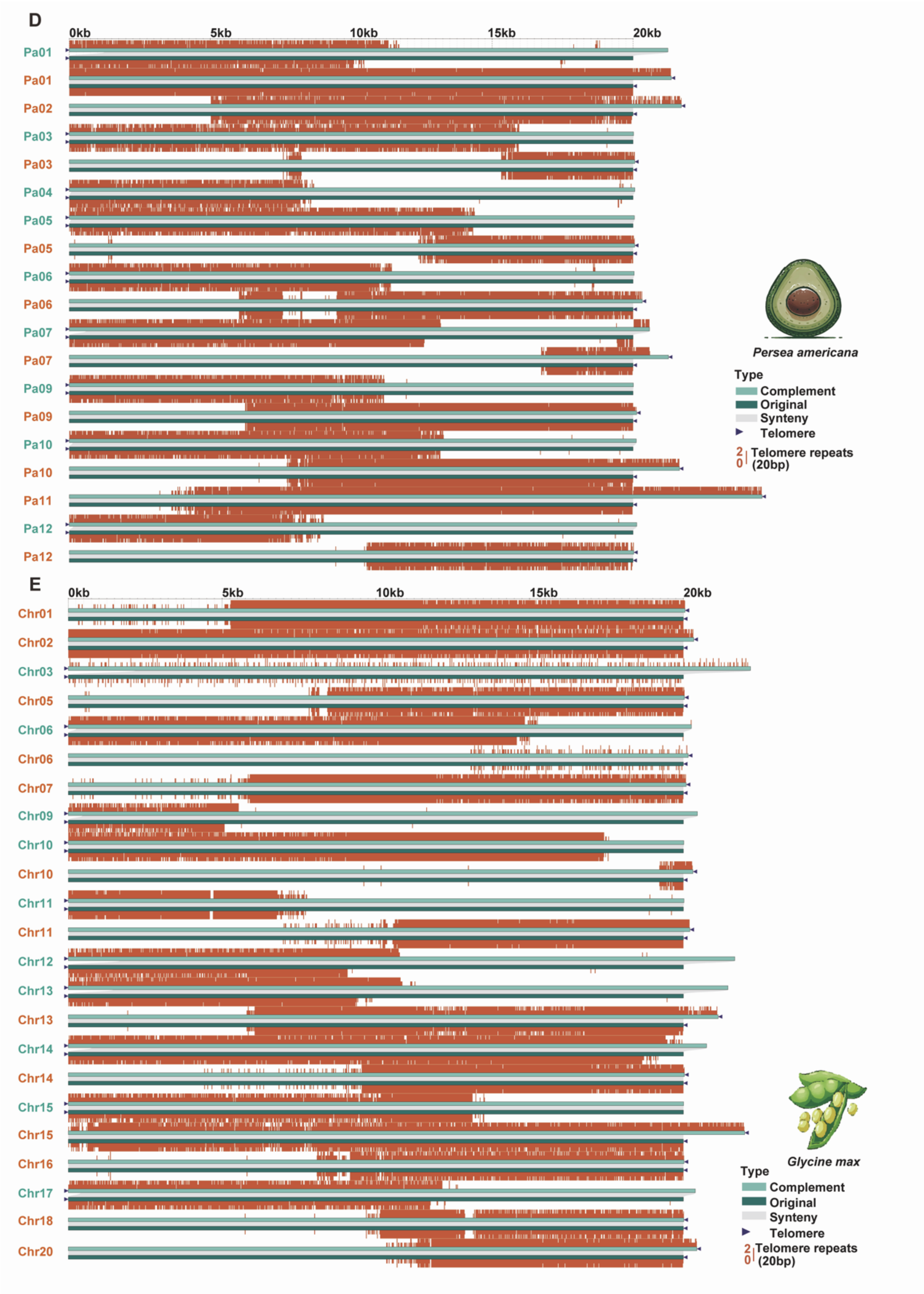

Supplemental Figure 10. Syntenic comparison between the genomes of five T2T assemblies with complemented telomeres (top) and the original genomes (bottom). Both genomes share a 20 kb overlapping region, with the regions exceeding 20 kb representing the supplemented telomeres. This figure specifically illustrates the synteny between the chromosome ends with supplemented telomeres and their corresponding chromosome

ends in the original genome for the following species: Rhodomyrtus tomentosa (A), Vigna unguiculata (B), Manihot esculenta (C), Persea americana (D), and Glycine max (E).

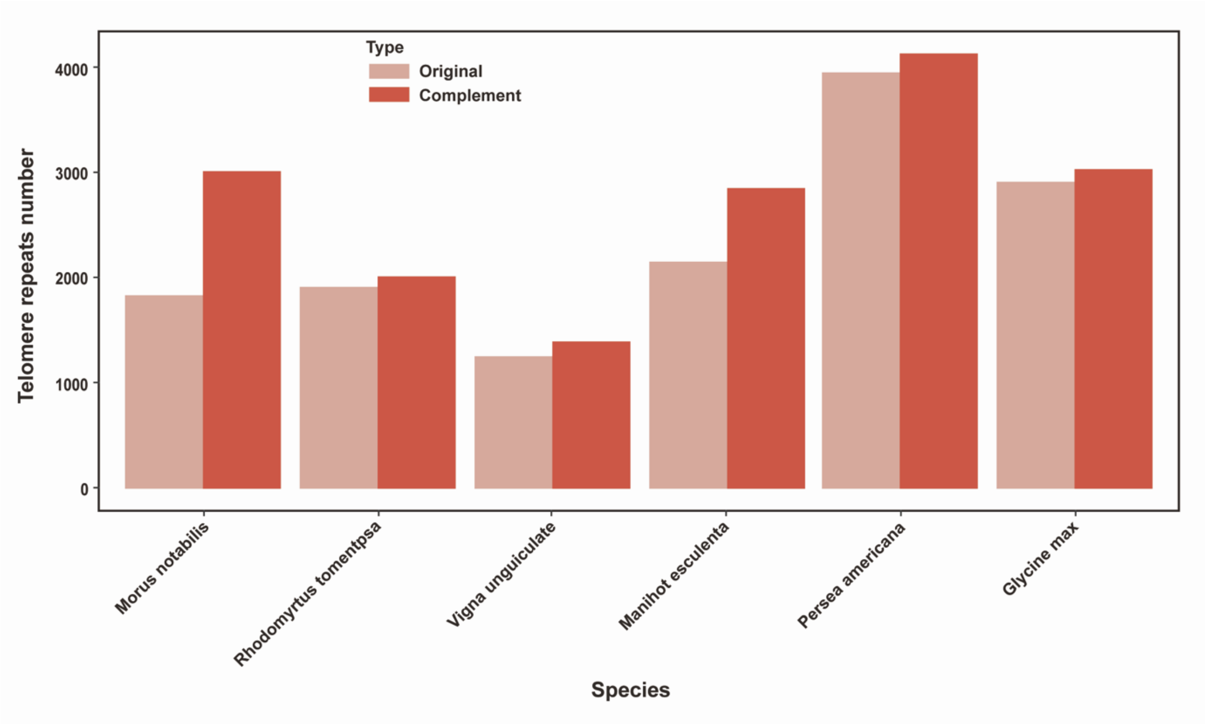

Supplemental Figure 11. The number of telomere repeats in six angiosperm T2T genomes before and after telomere supplementation was counted. For the original genome, the number of telomere repeats was calculated from the 100 kb region at the end of the chromosome; for the complemented genome, the number of telomere repeats was calculated from the 100 kb region of the original genome plus the supplemented region.

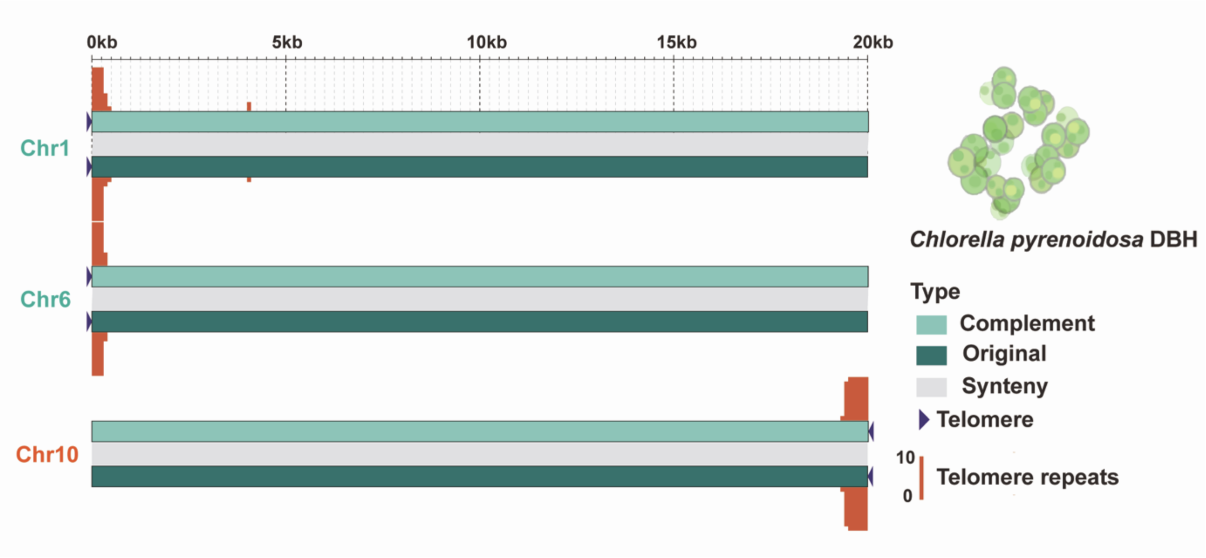

Supplemental Figure 12. Collinearity alignment before and after telomere supplementation in Chlorella pyrenoidosa DBH. The complemented telomeres (top) and the original genomes (bottom) share a 20 kb sequence segment, and the regions longer than 20 kb represent the telomeres supplemented by TeloComp. Green mark represents the left end of the chromosome, and orange mark represents right. The same applies to the following figures.

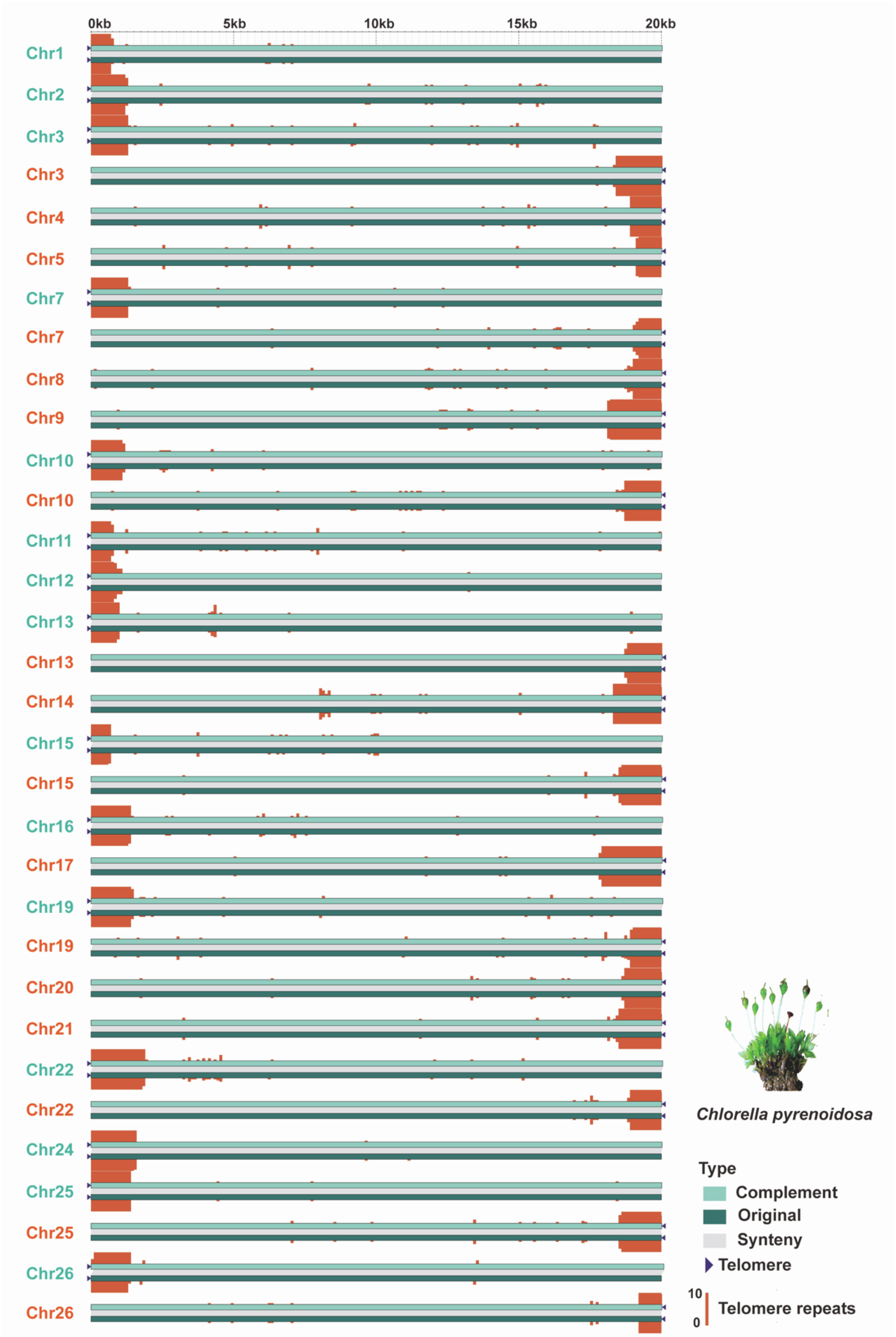

Supplemental Figure 13. Collinearity alignment before and after telomere supplementation in Physcomitrella patens. The complemented telomeres (top) and the original genomes (bottom) share a 20 kb sequence segment, and the regions longer than 20 kb represent the telomeres supplemented by TeloComp.

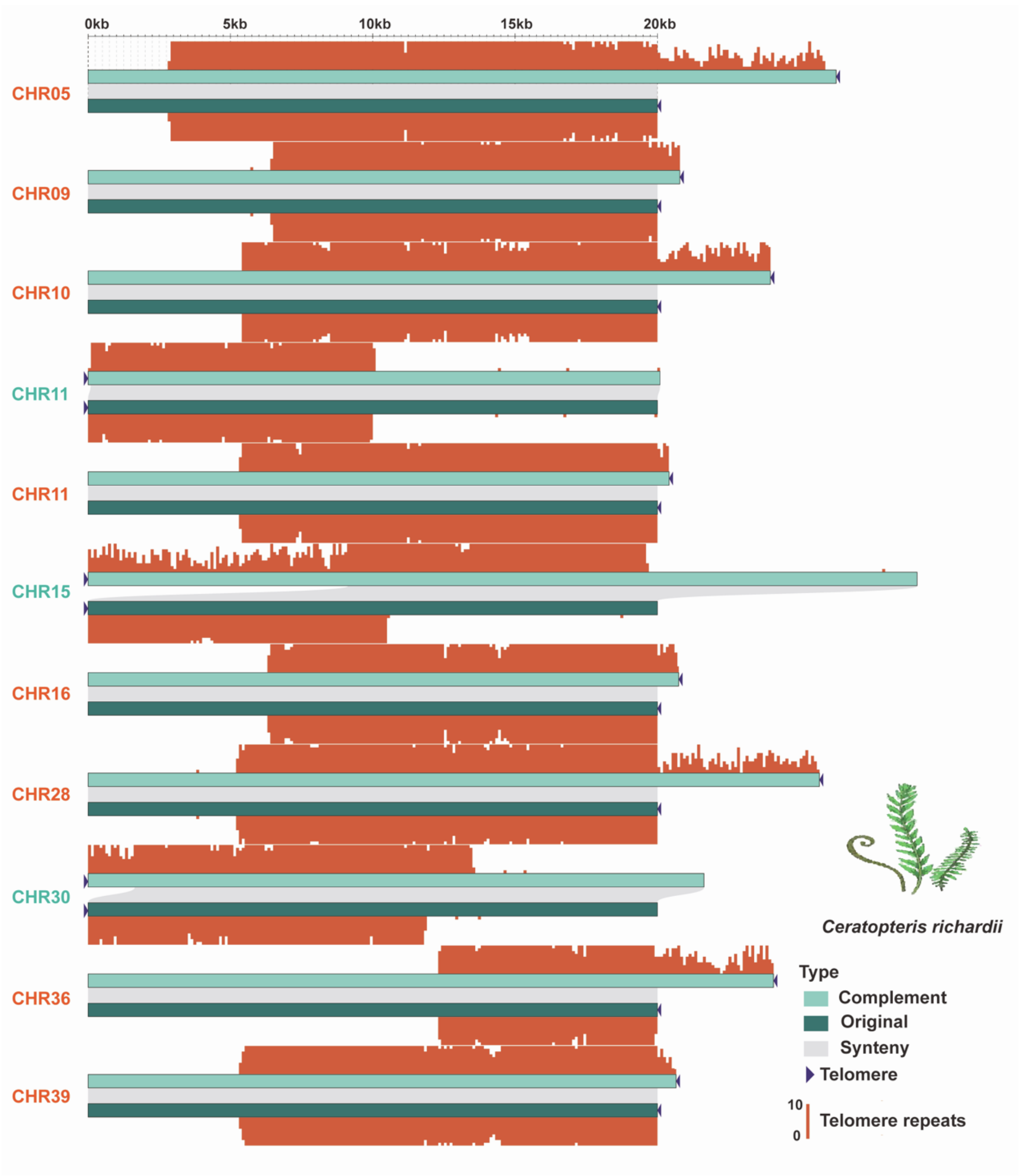

Supplemental Figure 14. Collinearity alignment before and after telomere supplementation in Chlamydomonas reinhardtii. The complemented telomeres (top) and the original genomes (bottom) share a 20 kb sequence segment, and the regions longer than 20 kb represent the telomeres supplemented by TeloComp.

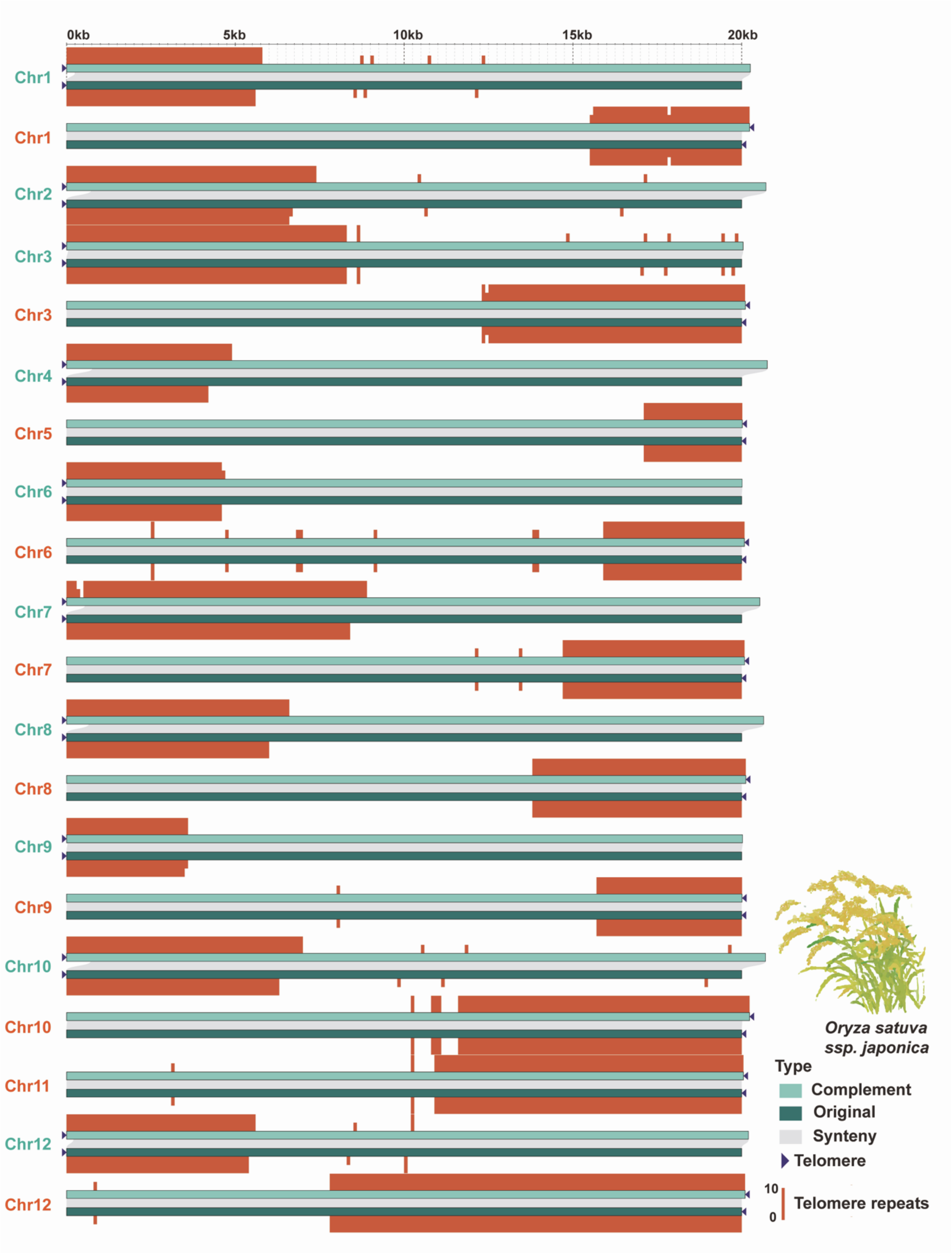

Supplemental Figure 15. Collinearity alignment before and after telomere supplementation in Oryza sativa ssp. japonica (Nipponbare). The complemented telomeres (top) and the original genomes (bottom) share a 20 kb sequence segment, and the regions longer than 20 kb represent the telomeres supplemented by TeloComp.

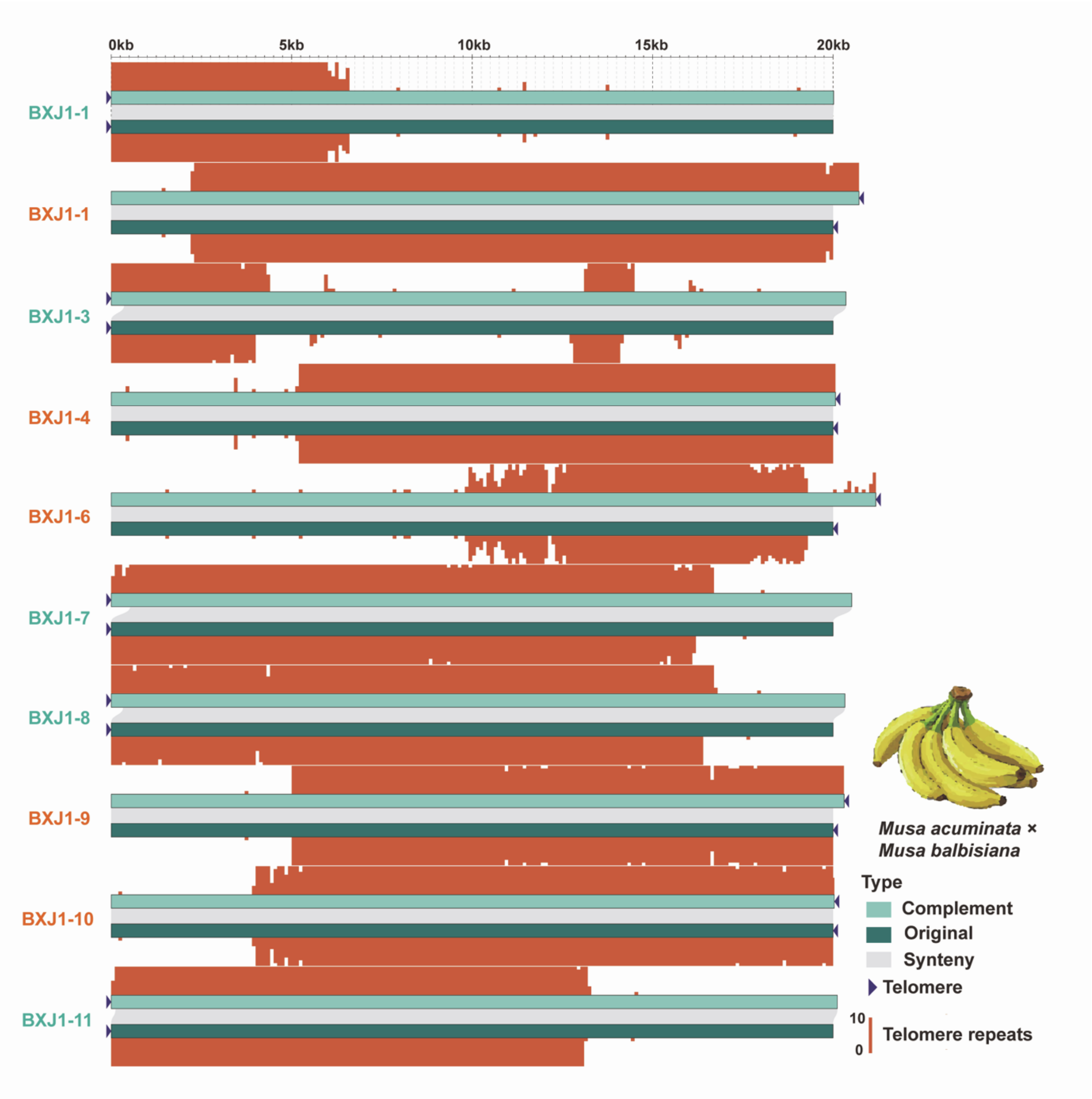

Supplemental Figure 16. Collinearity alignment before and after telomere supplementation in Musa acuminata × Musa balbisiana (triploid banana). The complemented telomeres (top) and the original genomes (bottom) share a 20 kb sequence segment, and the regions longer than 20 kb represent the telomeres supplemented by TeloComp.

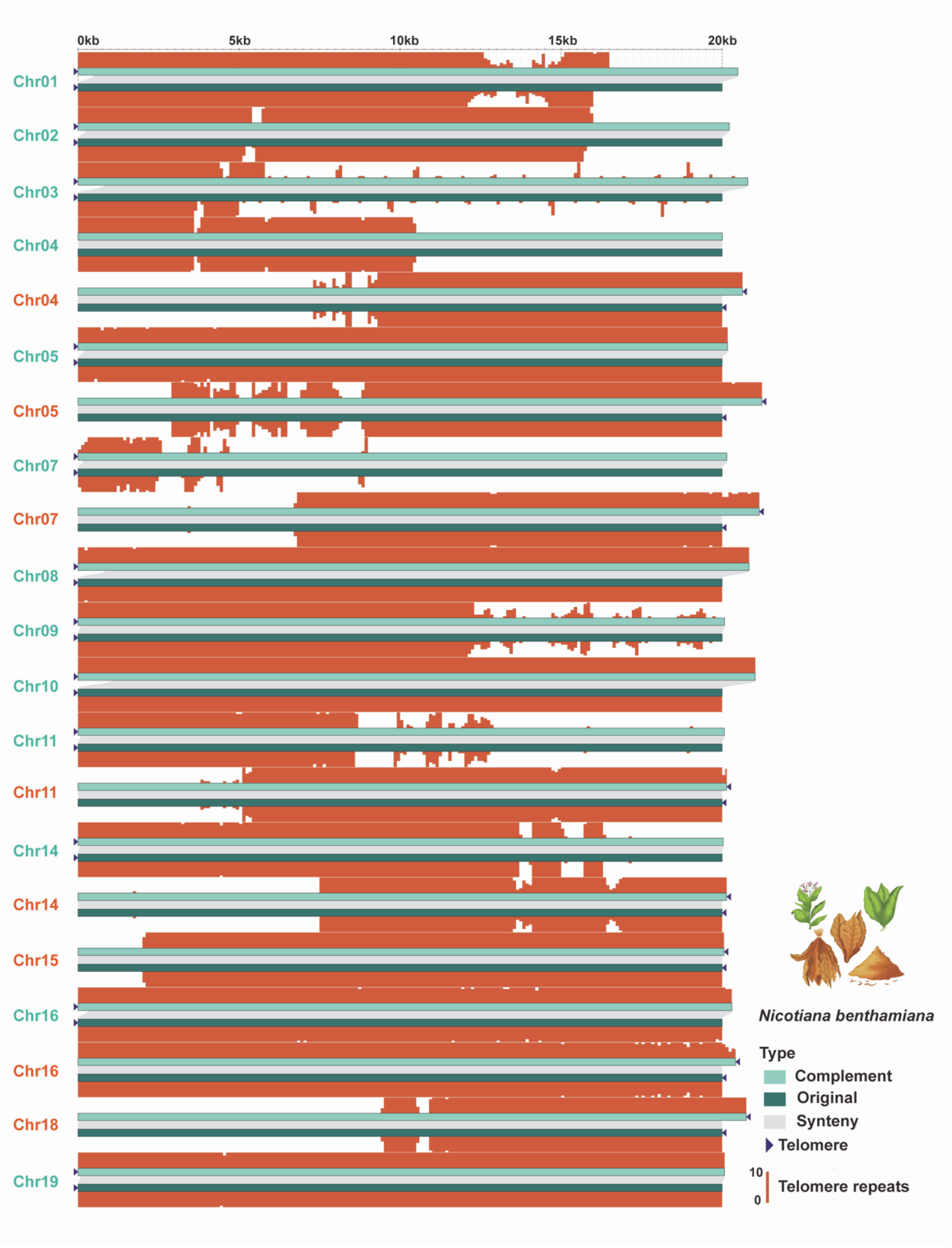

Supplemental Figure 17. Collinearity alignment before and after telomere supplementation in Nicotiana benthamiana (tetraploid). The complemented telomeres (top) and the original genomes (bottom) share a 20 kb sequence segment, and the regions longer than 20 kb represent the telomeres supplemented by TeloComp.

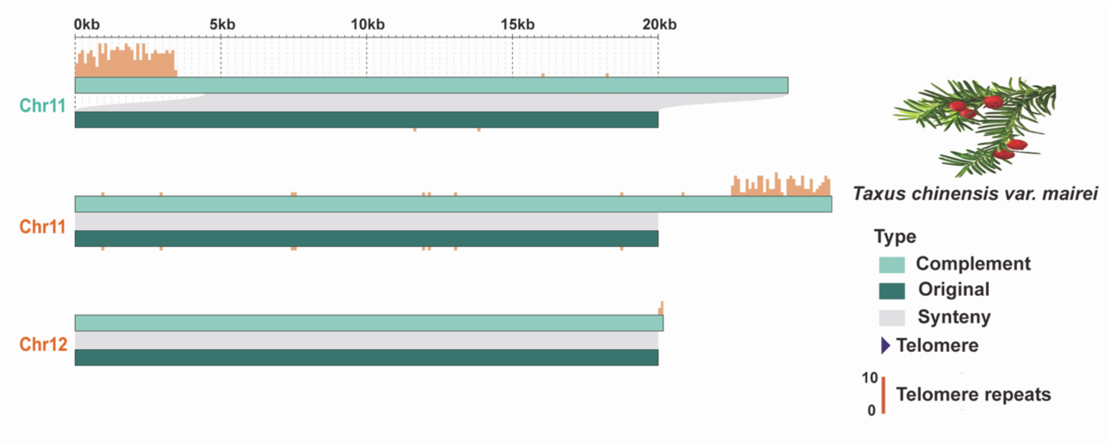

Supplemental Figure 18. Collinearity alignment before and after telomere supplementation in Taxus chinensis var. mairei. The complemented telomeres (top) and the original genomes (bottom) share a 20 kb sequence segment, and the regions longer than 20 kb represent the telomeres supplemented by TeloComp.

Supplemental tables

Supplemental Table1. Functional comparison between TeloComp and other telomere analysis tools.

Type	TeloComp	teloclip	TIDK	quarTeT	edgeCase	TelomereHunter
Species	Plant & Animal	Plant & Animal	Plant & Animal	Plant & Animal	Human	Human
Filter extend reads	Yes	Yes	No	No	No	No
Telomere identification	Yes	No	Yes	Yes	Yes	Yes
Telomere assembly	Yes	No	No	No	No	No
Telomere complement	Yes	No	No	No	No	No
Telomere visualization	Yes	No	Yes	Yes	Yes	Yes

Supplemental Table2. The size of the selected six T2T genome data and the corresponding TeloComp run consumption statistics.

Species	Genome size (Mb)	ONT (Gb)	HiFi (Gb)	User time (h)	Memory consumption (Gb)
Morus notabilis	398	43.1	15.06	2.36	50.14
Rhodomyrtus tomentpsa	470.35	33.4	11.02	3.49	43.63
Vigna unguiculate	521.3	72.1	30.7	43.56	81.19
Manihot esculenta	664.5	23	38	10.48	82.87
Persea americana	864	39.3	70.9	7.74	83.67
Glycine max	972.6	117.47	67.70	19.45	83.94

Supplemental Table3. Telomere information statistics of the six T2T genome supplements and completions.

Species	Chromosome	Original	Complement	Type	Length(bp)
Morus notabilis	chr2_L	1480	1498	CCCTAAA	141
	chr2_R	11	2688	TTTAGGG	24794
	chr3_L	1832	3009	CCCTAAA	27306
	chr3_R	991	1227	TTTAGGG	7635
	chr4_L	368	431	CCCTAAA	481
	chr5_L	683	699	CCCTAAA	131
	chr5_R	1786	1931	TTTAGGG	1300
	chr6_L	979	1222	CCCTAAA	1804
Rhodomyrtus tomentpsa	Chr3_R	346	399	TTTAGGG	1275
	Chr5_R	1425	1435	TTTAGGG	78
	Chr6_L	1347	1468	CCCTAAA	913
	Chr7_R	571	608	TTTAGGG	980
	Chr10_R	1915	2021	TTTAGGG	931
	Chr11_L	1776	1800	CCCTAAA	238
Vigna unguiculate	Vu03_L	1037	1039	CCCTAAA	23
	Vu03_R	39	44	TTTAGGG	131
	Vu04_L	219	234	CCCTAAA	154
	Vu05_R	673	746	TTTAGGG	680
	Vu06_R	31	38	TTTAGGG	423
	Vu07_L	350	579	CCCTAAA	1784
	Vu08_L	1250	1405	CCCTAAA	2746
	Vu11_R	950	951	TTTAGGG	10
	Chr1_L	480	1200	CCCTAAA	6345
	Chr2_L	1788	1860	CCCTAAA	1103
	Chr4_L	179	864	CCCTAAA	7741
	Chr4_R	5	498	TTTAGGG	25433
	Chr7_L	505	1366	CCCTAAA	6603
	Chr7_R	2148	2744	TTTAGGG	5499
	Chr8_L	289	1479	CCCTAAA	10575
	Chr9_L	524	950	CCCTAAA	7604

	Chr10_R	1627	1723	TTTAGGG	770
	Chr12_L	902	1181	CCCTAAA	10499
	Chr14_L	1203	1531	CCCTAAA	2967
Manihot esculenta	Chr14_R	234	706	TTTAGGG	10848
	Chr15_L	127	328	CCCTAAA	7971
	Chr16_R	638	1718	TTTAGGG	8836
	Chr17_L	1968	2757	CCCTAAA	6358
	Chr17_R	224	588	TTTAGGG	14405
	Chr18_L	33	448	CCCTAAA	17680
	Pa01_L	1384	1539	CCCTAAA	1237
	Pa01_R	3960	4135	TTTAGGG	1358
	Pa02_R	2066	2218	TTTAGGG	1714
	Pa03_L	1991	1992	CCCTAAA	17
	Pa03_R	725	731	TTTAGGG	60
	Pa04_L	1089	1096	CCCTAAA	64
	Pa05_L	1849	1855	CCCTAAA	48
	Pa05_R	1034	1039	TTTAGGG	56
	Pa06_L	1510	1514	CCCTAAA	40
	Pa06_R	1752	1789	TTTAGGG	328
	Pa07_L	2004	2081	CCCTAAA	586
	Pa07_R	426	505	TTTAGGG	1257
Persea americana	Pa09_L	1489	1490	CCCTAAA	12
	Pa09_R	1942	1947	TTTAGGG	128
	Pa10_L	1803	1817	CCCTAAA	113
	Pa10_R	1864	2071	TTTAGGG	1647
	Pa11_R	2248	2849	TTTAGGG	4576
	Pa12_L	1145	1162	CCCTAAA	130
	Pa12_R	1276	1278	TTTAGGG	27
	Chr01_R	2083	2089	TTTAGGG	52
	Chr02_R	2917	2954	TTTAGGG	337
	Chr03_L	1519	1563	CCCTAAA	2182
	Chr05_R	1528	1532	TTTAGGG	36
	Chr06_L	1970	2002	CCCTAAA	259
	Chr06_R	258	264	TTTAGGG	159
	Chr07_R	2076	2086	TTTAGGG	91
	Chr09_L	635	682	CCCTAAA	458
	Chr10_L	2372	2373	CCCTAAA	15
	Chr10_R	107	147	TTTAGGG	311

	Chr11_L	1007	1009	CCCTAAA	21
	Chr11_R	1477	1502	TTTAGGG	211
Glycine max	Chr12_L	1228	1422	CCCTAAA	1673
	Chr13_L	1338	1511	CCCTAAA	1452
	Chr13_R	1915	2015	TTTAGGG	1131
	Chr14_L	2609	2711	CCCTAAA	755
	Chr14_R	1612	1615	TTTAGGG	36
	Chr15_L	1668	1668	CCCTAAA	9
	Chr15_R	2782	3038	TTTAGGG	1991
	Chr16_R	1478	1479	TTTAGGG	22
	Chr17_L	1514	1559	CCCTAAA	390
	Chr18_R	1283	1284	TTTAGGG	14
	Chr20_R	1260	1310	TTTAGGG	436

Note: In the "Chromosome" column, chromosome identifiers consist of the chromosome number and the direction, where "L" denotes the left end and "R" denotes the right end, indicating the specific chromosome terminus where telomere supplementation was conducted.

Supplemental Table4. Statistics of test genome datasets (HiFi, ONT, and WGS) from algae, mosses, ferns, gymnosperms, monocots, and polyploid plants used for TeloComp evaluation.

Species	Genome (Mb)	ONT (Gb)	HiFi (Gb)	WGS (Gb)
Chlorella pyrenoidosa DBH	53.41	22.12	23.74	-
Physcomitrium patens	464.03	42.98	85.99	51.14
Ceratopteris richardii	7,669.76	64.83	-	590.32
Oryza satuva ssp. japonica	385.70	159.90	159.70	68.00
Musa acuminata × *Musa balbisiana*	477.16	48.00	102.06	44.13
Nicotiana benthamiana	2,849.30	136.50	332.60	886.00
Taxus chinensis var. mairei	10,4475.52	-	318.05	693.73

Supplemental Table 5. Genome data sources from algae, mosses, ferns, gymnosperms, monocots, and polyploid plants used for TeloComp.

Species	Raw data accession	Reference
Chlorella pyrenoidosa DBH	CRA007945	Wang, Bo, et al. (2024)
Physcomitrium patens	PRJCA032473	Bi, Guiqi, et al. (2024)
Ceratopteris richardii	PRJNA729743	Marchant, D. Blaine, et al. (2022)
Oryza satuva ssp. japonica	PRJCA018610	Shang, Lianguang, et al. (2023)
Musa acuminata×Musa balbisiana	PRJCA016940	Huang, Hui-Run, et al. (2023)
Nicotiana benthamiana	PRJCA022857	Guo, Li, et al. (2024)
Taxus chinensis var. mairei	PRJCA001755	Liu, H. L. et al. (2021)

Supplemental Table 6. The statistical information on the number and length of the supplemented telomeres in the genomes of various plant groups.

Species	Chromosome	Original	Complement	Type	Length(bp)
Chlorella pyrenoidosa DBH	Chr1_L	43	47	CCCTAAA	22
	Chr6_L	39	42	CCCTAAA	15
	Chr10_R	81	83	TTTAGGG	19
Physcomitrium patens	Chr1_L	105	108	CCCTAAA	31
	Chr2_L	184	190	CCCTAAA	39
	Chr3_L	205	206	CCCTAAA	17
	Chr3_R	229	230	TTTAGGG	36
	Chr4_R	168	169	TTTAGGG	10
	Chr5_R	133	135	TTTAGGG	19

	Chr7_L	187	189	CCCTAAA	27
	Chr7_R	136	137	TTTAGGG	12
	Chr8_R	165	168	TTTAGGG	27
	Chr9_R	275	276	TTTAGGG	15
	Chr10_L	171	173	CCCTAAA	29
	Chr10_R	198	199	TTTAGGG	8
	Chr11_L	122	123	CCCTAAA	13
	Chr12_L	130	132	CCCTAAA	12
	Chr13_L	149	153	CCCTAAA	27
	Chr13_R	173	176	TTTAGGG	30
	Chr14_R	258	259	TTTAGGG	15
	Chr15_L	108	113	CCCTAAA	41
	Chr15_R	212	214	TTTAGGG	26
	Chr16_L	208	214	CCCTAAA	48
	Chr17_R	301	305	TTTAGGG	33
	Chr19_L	211	219	CCCTAAA	55
	Chr19_R	180	173	TTTAGGG	12
	Chr20_R	202	203	TTTAGGG	17
	Chr21_R	223	224	TTTAGGG	20
	Chr22_L	281	286	CCCTAAA	46
	Chr22_R	167	168	TTTAGGG	14
	Chr24_L	225	229	CCCTAAA	25
	Chr25_L	200	201	CCCTAAA	14
	Chr25_R	219	220	TTTAGGG	16
	Chr26_L	190	193	CCCTAAA	91
	Chr26_R	121	123	TTTAGGG	23
Ceratopteris richardii	CHR05_R	2352	2642	TTAGGG	6282
	CHR09_R	1889	1961	TTAGGG	792
	CHR10_R	1962	2266	TTAGGG	3973
	CHR11_L	1362	1364	CCCTAA	92
	CHR11_R	2016	2050	TTAGGG	413
	CHR15_L	1460	2053	CCCTAA	9128
	CHR16_R	1854	1923	TTAGGG	749
	CHR28_R	2010	2295	TTAGGG	5694

	CHR30_L	1587	1731	CCCTAA	1638
	CHR36_R	1006	1266	TTAGGG	4082
	CHR39_R	1978	2014	TTAGGG	659
Oryza satuva ssp. japonica	Chr1_L	772	806	CCCTAA	259
	Chr1_R	397	428	TTAGGG	233
	Chr2_L	934	996	CCCTAA	718
	Chr3_L	1147	1149	CCCTAA	44
	Chr3_R	1051	1060	TTAGGG	112
	Chr4_L	484	593	CCCTAA	761
	Chr5_R	305	307	TTAGGG	20
	Chr6_L	645	647	CCCTAA	16
	Chr6_R	580	589	TTAGGG	83
	Chr7_L	1150	1159	CCCTAA	540
	Chr7_R	724	735	TTAGGG	84
	Chr8_L	824	904	CCCTAA	653
	Chr8_R	860	876	TTAGGG	123
	Chr9_L	490	493	CCCTAA	27
	Chr9_R	495	496	TTAGGG	12
	Chr10_L	860	943	CCCTAA	707
	Chr10_R	1193	1202	TTAGGG	234
	Chr11_R	1282	1289	TTAGGG	53
	Chr12_L	758	769	CCCTAA	204
	Chr12_R	1576	1588	TTAGGG	102
Musa acuminata × *Musa balbisiana*	BXJ1-1_L	874	875	CCCTAAA	15
	BXJ1-1_R	2512	2602	TTTAGGG	719
	BXJ1-3_L	741	788	CCCTAAA	356
	BXJ1-4_R	2103	2110	TTTAGGG	67
	BXJ1-6_R	1026	1044	TTTAGGG	1186
	BXJ1-7_L	2244	2292	CCCTAAA	519
	BXJ1-8_L	2273	2313	CCCTAAA	329
	BXJ1-9_R	1896	1936	TTTAGGG	307
	BXJ1-10_R	2038	2040	TTTAGGG	35
	BXJ1-11_L	1842	1849	CCCTAAA	120

Nicotiana benthamiana	Chr01_L	1930	1992	CCCTAAA	493
	Chr02_L	2148	2178	CCCTAAA	226
	Chr03_L	747	861	CCCTAAA	805
	Chr04_L	1366	1368	CCCTAAA	12
	Chr04_R	1558	1643	TTTAGGG	636
	Chr05_L	2756	2777	CCCTAAA	175
	Chr05_R	1999	2159	TTTAGGG	1238
	Chr07_L	351	355	CCCTAAA	152
	Chr07_R	1845	1970	TTTAGGG	1158
	Chr08_L	2810	2911	CCCTAAA	840
	Chr09_L	1861	1871	CCCTAAA	79
	Chr10_L	2842	2964	CCCTAAA	1032
	Chr11_L	1320	1328	CCCTAAA	76
	Chr11_R	2053	2063	TTTAGGG	145
	Chr14_L	2062	2067	CCCTAAA	42
	Chr14_R	1649	1669	TTTAGGG	143
	Chr15_R	2561	2567	TTTAGGG	60
	Chr16_L	2676	2711	CCCTAAA	308
	Chr16_R	2698	2727	TTTAGGG	420
	Chr18_R	1432	1535	TTTAGGG	753
	Chr19_L	2748	2758	CCCTAAA	80
Taxus chinensis var. mairei	Chr11_L	2	273	CCCTAAA	4458
	Chr11_R	8	137	TTTAGGG	5960

Note: In the "Chromosome" column, the names are composed of the chromosome number and direction, where "L" represents the left end of the chromosome and "R" represents the right end, indicating the chromosome end where supplemented telomeres are located.

Supplemental references

Danecek, P., Bonfield, J.K., Liddle, J., Marshall, J., Ohan, V., Pollard, M.O., Whitwham, A., Keane, T., McCarthy, S.A., Davies, R.M., et al. (2021). Twelve years of SAMtools and BCFtools. GigaScience **10**:10. 10.1093/gigascience/giab008.

Hu, J., Fan, J., Sun, Z., and Liu, S. (2019). NextPolish: a fast and efficient genome polishing tool for long-read assembly. Bioinformatics **36**:2253-2255. 10.1093/bioinformatics/btz891.

Kolmogorov, M., Yuan, J., Lin, Y., and Pevzner, P.A. (2019). Assembly of long, error-prone reads using repeat graphs. Nature Biotechnology **37**:540-546. 10.1038/s41587-019-0072-8.

Li, H. (2018). Minimap2: pairwise alignment for nucleotide sequences. Bioinformatics **34**:3094-3100. 10.1093/bioinformatics/bty191.

Li, H., and Durbin, R. (2009). Fast and accurate short read alignment with Burrows–Wheeler transform. Bioinformatics **25**:1754-1760. 10.1093/bioinformatics/btp324.

Walker, B.J., Abeel, T., Shea, T., Priest, M., Abouelliel, A., Sakthikumar, S., Cuomo, C.A., Zeng, Q., Wortman, J., and Young, S.K. (2014). Pilon: an integrated tool for comprehensive microbial variant detection and genome assembly improvement. PloS one **9**:e112963.

Zhou, Z.W., Yu, Z.G., Huang, X.M., Liu, J.S., Guo, Y.X., Chen, L.L., and Song, J.M. (2022). GenomeSyn: a bioinformatics tool for visualizing genome synteny and structural variations. J Genet Genomics **49**:1174-1176. 10.1016/j.jgg.2022.03.013.
